# The hepatocyte growth factor/mesenchymal epithelial transition factor axis in high-risk pediatric solid tumors and the anti-tumor activity of targeted therapeutic agents

**DOI:** 10.3389/fped.2022.910268

**Published:** 2022-08-10

**Authors:** Megan Grundy, Aru Narendran

**Affiliations:** ^1^Cumming School of Medicine, University of Calgary, Calgary, AB, Canada; ^2^POETIC Laboratory for Preclinical and Drug Discovery Studies, Division of Pediatric Oncology, Alberta Children’s Hospital, University of Calgary, Calgary, AB, Canada

**Keywords:** mesenchymal epithelial transition factor (MET), hepatocyte growth factor (HGF), scatter factor, pediatric solid tumor, metastatic, targeted therapy

## Abstract

Clinical trials completed in the last two decades have contributed significantly to the improved overall survival of children with cancer. In spite of these advancements, disease relapse still remains a significant cause of death in this patient population. Often, increasing the intensity of current protocols is not feasible because of cumulative toxicity and development of drug resistance. Therefore, the identification and clinical validation of novel targets in high-risk and refractory childhood malignancies are essential to develop effective new generation treatment protocols. A number of recent studies have shown that the hepatocyte growth factor (HGF) and its receptor Mesenchymal epithelial transition factor (c-MET) influence the growth, survival, angiogenesis, and metastasis of cancer cells. Therefore, the c-MET receptor tyrosine kinase and HGF have been identified as potential targets for cancer therapeutics and recent years have seen a race to synthesize molecules to block their expression and function. In this review we aim to summarize the literature that explores the potential and biological rationale for targeting the HGF/c-MET pathway in common and high-risk pediatric solid tumors. We also discuss selected recent and ongoing clinical trials with these agents in relapsed pediatric tumors that may provide applicable future treatments for these patients.

## Introduction

Mesenchymal epithelial transition factor (c-MET) is a cell-surface receptor tyrosine kinase that is widely expressed throughout many organ systems. c-MET is predominantly expressed in epithelial cells, while its ligand (hepatocyte growth factor–HGF) is largely expressed by cells of mesenchymal origin (1, 2). Under normal conditions, this signaling pathway plays a crucial role in embryogenesis, wound-healing and regeneration, but has been identified as a driver of cancerous growth, metastasis, and drug resistance (3–5).

The c-MET receptor tyrosine kinase was first discovered as a result of work done to isolate a transforming gene chemically induced in human osteosarcoma cells (6–8). The ligand HGF [or scatter factor (SF) (9)] was later identified as the ligand for the c-MET receptor (10). The HGF/c-MET signaling pathway is complex [reviewed in (11)], and promotes diverse cellular activities including survival, proliferation, motility, and differentiation (12, 13). The HGF/c-MET pathway is crucial during embryogenesis; mice in which either the *MET* or *HGF* gene is mutated do not survive *in utero*. Disruption of the HGF/c-MET pathway results in impaired placentation, liver development, and development of the skeletal muscles of the limbs (14–16). In adult organisms, the HGF/c-MET pathway has been shown to drive wound-healing and regeneration. Increased plasma levels of HGF are seen after various insults including ischemic injury to the myocardium (17, 18), acute renal failure (19), partial hepatectomy (20), and acute lung injury (21). Conditional mutation of the *MET* gene in mice greatly impairs liver regeneration after injury or partial hepatectomy (20, 22, 23), skin regeneration and wound healing (24), and renal protection following acute kidney injury (25).

Binding of activated HGF to the c-MET cell surface receptor induces receptor dimerization which activates the tyrosine kinase through phosphorylation of two tyrosine residues in the kinase activation loop. This in turn causes autophosphorylation of tyrosine residues in the carboxy-terminal substrate-binding tail of the cytoplasmic MET protein. Here, a wide number of cytoplasmic effector proteins are recruited including phosphoinositide 3 kinase (PI3K), Src homology-2-containing (SHC), Src, Src homology 2 domain-containing phosphatase-2 (SHP2), signal transducer and activator of transcription 3 (STAT3), growth factor receptor-bound protein 2 (GRB2), and GRB2-associated binding protein 1 (GAB1) (26–29). Notably, phosphorylated GAB1 bound to MET (directly or through GRB2) provides additional binding sites for cytoplasmic effector proteins (30, 31). Important signaling cascades activated downstream from c-MET include the RAS-MAPK and PI3K-Akt pathways, which affect transcription ultimately leading to increased cell cycle progression, proliferation, and motility [reviewed in (11)]. Other pathways affected by c-MET signaling–such as RAC-CDC42, p21-activated kinase (P21), and RAP1-FAK–work in the cytoplasm or at the plasma membrane to cause cytoskeleton changes, and reduce cellular adhesion, ultimately promoting cell motility and migration (4).

In addition to the “main” HGF/c-MET signaling pathway, there is evidence for significant crosstalk between c-MET and other cell surface proteins, including integrins, plexins, CD44, and other receptor tyrosine kinases, notably the epidermal growth factor receptor (EGFR) (32, 33). These cross-receptor interactions (often amplified in cancer) have been shown to play a key role in acquired resistance to chemotherapy targeting growth receptors, such as EGFR (34, 35).

Finally, c-MET can be downregulated by degradation after activation. The c-MET receptor is internalized through clathrin-mediated endocytosis and is either ubiquitinated and degraded or recycled back to the cell surface. Ubiquitination is mediated by binding of Cbl (an E3 ubiquitin ligase) to the phosphorylated tail of c-MET, and disruption of this interaction can lead to increased activity of c-MET (36–39). Other mechanisms for regulation of HGF/c-MET signaling include negative feedback by protein kinase C (PKC) (40, 41), degradation of the c-MET receptor by extracellular metalloproteases (42), and dephosphorylation of c-MET tyrosine residues in the activation or docking domains by intracellular phosphatases (43–45).

A more thorough discussion of the molecular signaling underpinning the HGF/c-MET pathway is beyond the scope of this review, and is summarized schematically in (5, 46).

## Hepatocyte growth factor/mesenchymal epithelial transition factor axis and cancer

**FIGURE 1:**
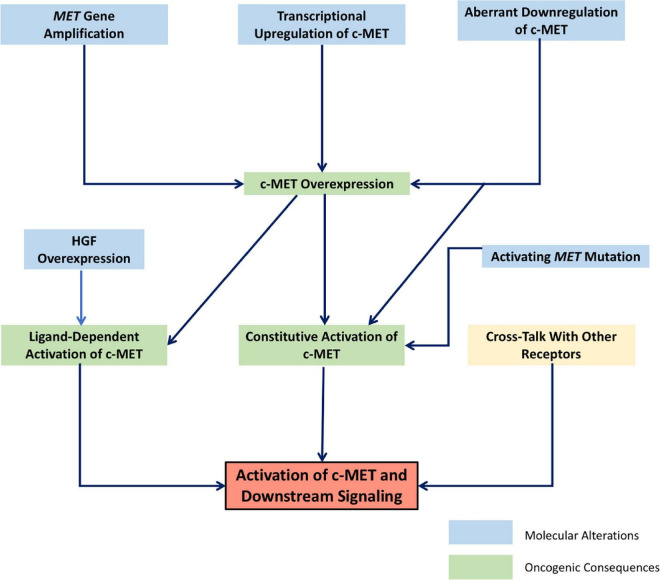
Schematic representation of the molecular mechanisms leading to dysregulated signaling of the HGF/c-MET pathway. Interrelated molecular factors lead to changes in HGF/c-MET expression and activity, resulting in the activation of downstream signaling pathways which promote oncogenic cellular changes.

Currently, there is a large body of literature linking the HGF/c-MET pathway to cancer (46). Indeed, the c-MET gene was first identified as a protooncogene transformed by its association with translocated promoter region (TPR), leading to constitutive activation and oncogenic changes (6–8, 47). This same activating TPR-MET oncogene has since been identified in human gastric cancer (48). There are many ways in which the HGF/c-MET pathway–which, as discussed above, is crucial in normal physiology for cell survival, proliferation, motility and migration–can contribute to a progression toward uncontrolled cell growth and invasiveness. Changes that lead to increased levels of active HGF, c-MET expression, c-MET activation, or to decreased c-MET downregulation and degradation generally contribute to a more oncogenic phenotype. While genetic changes such as activating point mutations, or amplification of the *MET* gene have been identified in a wide range of human cancers, increased activity of the HGF/c-MET pathway is often seen in cancer as a result of physical and chemical changes in the tumor stroma, leading to upregulation of c-MET, or clonal selection of c-MET-expressing cancer cells. The result is often a subset of invasive cancer cells resistant to chemotherapy, and prone to dissemination (46). Figure 1 shows schematically the broad categories of molecular alterations that can contribute to dysregulated HGF/c-MET signaling in cancer.

In the following, the role of the HGF/c-MET pathway in pediatric solid cancers, as is currently understood in the literature, will be reviewed.

## Pediatric malignancies

### Rhabdomyosarcoma

Rhabdomyosarcoma (RMS) is the most common soft-tissue sarcoma in pediatric patients (49). The majority of pediatric RMS can be classified histologically as either “embryonal” (ERMS) or “alveolar” (ARMS) (50). ARMS generally has a worse prognosis than ERMS, is more prone to metastasis, and is more often diagnosed at a later stage (51, 52). In the majority of ARMS tumors, a chromosomal translocation t(2;13) is identified, which leads to the expression of fusion proteins PAX3-FOXO1 or (less frequently) PAX7-FOXO1 [t(1;13)] (50). These fusion proteins combine the PAX3/7 binding domain with the FOXO1 activation domain (aka FOXO1A), resulting in a transcription activator more powerful than PAX3 or PAX7 alone (53). Clinical research indicates that the presence of a fusion protein (“fusion positive”) is a more important prognostic indicator than the histologic classification of ARMS vs. ERMS (54). Early research links the HGF/c-MET pathway to development of RMS; c-MET was identified as a target of the PAX3-FOXO1 fusion protein (55), and a murine model of RMS showed that dysregulation of HGF/c-MET signaling on the background of suppression of *INK4a/ARF* (locus that encodes two tumor suppressors) was sufficient to lead to the development of RMS tumors with a similar molecular signature to human RMS tumors (56).

*In vitro* studies of the relationship between c-MET and the PAX3-FOXO1 fusion protein have shown that while expression of c-MET is upregulated by PAX3-FOXO1, PAX3-FOXO1 expression is not necessary for c-MET expression and activity. Ginsberg et al. found that in myogenic cells transfected with PAX3-FOXO1 and fusion-positive ARMS cells, c-MET expression correlated with levels of PAX3-FOXO1. However, high levels of c-MET expression were also found in some samples of ERMS, suggesting that PAX3-FOXO1 is not necessary for c-MET expression (57). The correlation of c-MET upregulation and PAX3-FOXO1 was confirmed by Taulli et al., who also found that while PAX3-FOXO1 could induce oncogenic cell growth, this transformation was dependent on HGF/c-MET signaling. Notably, silencing of the c-MET receptor inhibited cell proliferation, and increased apoptosis in *both* ARMS- and the fusion-negative ERMS-derived cell lines, which underscores a role for the HGF/c-MET pathway in RMS beyond the effects of the PAX3-FOXO1 transcription activator found largely in ARMS. Indeed, when the c-MET receptor in ERMS xenografts was conditionally silenced with an anti-Met short hairpin RNA (shRNA), tumor growth stopped and subsequently regressed (58).

Particularly in ARMS, the presence of metastases at diagnosis confers a poor prognosis (51, 52). Given the physiological role for the HGF/c-MET pathway in cell motility and migration, particularly in the case of skeletal muscle and limb development (15), this pathway has been keenly studied with regards to its role in promoting metastasis of RMS. Jankowski et al. showed that in response to stimulation with HGF, c-MET-positive (assessed by immunostaining and flow cytometry) RMS cell lines undergo a number of pro-metastatic changes *in vitro* including increased locomotion, motility-related redistribution of the actin cytoskeleton, and increased cell migration. Notably, while HGF stimulation *in vitro* did not increase RMS cell proliferation, it did confer increased survival following exposure to radiation or cytotoxic chemotherapy, suggesting that HGF/c-MET might be an important target for therapy-resistant RMS (59). RMS cells were found to migrate toward HGF-rich conditioned medium from bone marrow fibroblasts, and *in vivo*, RMS cells were attracted into the bone marrow in a manner that was dependent on c-MET signaling. This effect was most pronounced for ARMS cell lines, which showed higher c-MET expression than ERMS cell lines (59). Downregulation of c-MET in RMS cell lines decreased both *in vivo* tumor growth and bone marrow engraftment (60). A clinical study examining samples from 40 patients with RMS found that in the subset of patients whose cancer had infiltrated the bone marrow (*n* = 16), the infiltrating cells had greater levels of c-MET expression than did cells from the corresponding primary tumors (61). These findings all implicate the HGF/c-MET pathway as a key driver of RMS invasive and metastatic spread.

RMS arises from dysregulated myogenic progression with loss of terminal cell differentiation. Lower c-MET expression has been correlated with greater levels of differentiation (62). In normal myogenic development, downregulation of c-MET is required for terminal differentiation (63, 64). It has been postulated that the sustained c-MET expression seen in RMS could result from decreased post-transcriptional downregulation by myomiRs (particularly miR-1 and miR-206, microRNA sequences which are physiologically upregulated during myogenesis). Low levels of miR-1 and miR-206 were found in ERMS and ARMS compared to normal muscle, and re-expression of these miRNAs was found to promote differentiation, and inhibit growth of RMS xenografts. c-MET was shown to be downregulated in myogenic precursor cells by miR-206 at the onset of myogenesis, and the expression of miR-206 in RMS cells was found to inhibit c-MET signaling (65, 66).

Histologically, cells from ERMS tumors have a more differentiated “spindle-shaped” morphology compared to the more aggressive small round cells found in ARMS tumors (50). When c-MET signaling was blocked in ARMS cells, they began to resemble ERMS cells morphologically (62). Conversely, when c-MET was constitutively activated (by introduction of the TPR-MET oncogene) ERMS cells did not differentiate. Control ERMS cells developed the typical histology of ERMS tumors with elongated spindle morphology, while TPR-MET-activated ERMS cells retained morphology typical of the more aggressive ARMS tumors. Xenograft tumors derived from the constitutively c-MET-activated ERMS cells were less differentiated, faster growing, had increased vascularization, and more distant metastases compared to controls. Though targeted activation of c-MET promoted these aggressive changes to the typical ERMS phenotype, tumors derived from ARMS cells still grew more aggressively, suggesting that factors other than c-MET activity contribute to the phenotype of ARMS (64).

To date, there are but a few studies linking c-MET activity to clinicopathological parameters and patient outcomes in RMS. Chen et al. examined levels of c-MET expression in primary RMS tumors, and while they identified expression of c-MET in all tumor samples, only a minority of tumors *over*expressed c-MET. Their analysis did, however, find a positive correlation between higher expression of c-MET and later staging of cancer, and the presence of a PAX3/7-FOXO1 fusion protein. Higher expression of c-MET was correlated with poorer survival (67). Diomedi-Camassei et al. also found a similar correlation between c-MET expression and advanced disease, however, this study did not identify a relationship between overall survival and c-MET expression (61). A more recent study by Du et al. found a significant effect of c-MET expression on overall survival only in ERMS or “fusion-negative” RMS, and though this study identified greater expression of c-MET in RMS samples compared to normal muscle controls, the difference between c-MET expression in ARMS and ERMS samples was not significant (68). Further analysis of larger cohorts of RMS patients would be beneficial to clarify the role (if any) of c-MET in RMS prognosis.

### Osteosarcoma

Osteosarcoma (OS) is the most common primary bone tumor, and occurs in a bimodal age distribution with a peak in the pediatric age range. Survival in OS has plateaued since the 1980’s, and new treatments are needed, especially for those with advanced disease (69). The c-MET gene was first identified in human OS cells that had been treated with a chemical carcinogen (7). A number of studies have since identified overexpression of c-MET in a large portion of OS tissue samples (though the rates of overexpression reported range substantially between studies) (70–73). Human OS-derived cell lines also have been shown to have increased (but variable) c-MET expression (74, 75), and invasive cell behavior (increased motility and invasion) after stimulation with HGF (74).

Patane et al. showed that overexpression of c-MET could transform osteoblasts (with undetectable c-MET levels at baseline) to an OS-like phenotype, capable of anchorage-independent growth, and increased basement membrane and 3-D invasion. Development of this OS-like phenotype was inhibited by blockade of c-MET receptor dimerization or with c-MET targeting shRNA (76). A more recent study from the same group showed that MET-OS “clones”— created from c-MET overexpression in normal bone-derived cells — originated from selective expansion of a subpopulation of committed osteoprogenitor cells at an early stage of differentiation, and that c-MET overexpression inhibited full osteoblast differentiation (77). These studies suggest a role for the HGF/c-MET pathway in the development of OS.

A number of similar reports have suggested a role for c-MET in the development of OS through suppression or downregulation of microRNAs (miRNAs). Specifically, studies have shown that a range of cancer-related miRNAs are decreased in OS, inversely correlated with increased c-MET expression, and directly target the wild-type 3′-UTR of the *MET* gene. Re-expression of these suppressed miRNAs has been shown to lead to reduced OS growth and invasion (78–84).

Pharmacological c-MET inhibition in OS has been shown to decrease malignant behavior of OS cells in culture, and to suppress *in vivo* xenograft tumor growth (85, 86). Fioramonti et al. showed that treatment with cabozantinib (an inhibitor of multiple RTKs including c-MET and VEGFR-2), in addition to directly reducing OS proliferation and migration, has an indirect inhibitory effect on RANK-expressing (a poor prognostic marker) OS cell lines by decreasing RANK-L production by osteoblasts (86).

### Ewing sarcoma

After OS, Ewing Sarcoma (ES) is the second most common primary pediatric bone tumor. Fleuren et al. found that a majority of samples of ES (including primary tumors, metastases and post-chemotherapy resections) had medium to high levels of cytoplasmic c-MET expression. More specifically, however, they found that expression of c-MET localized to the plasma membrane (and not cytoplasmic c-MET) was associated with worse overall survival. ES cell lines were susceptible to treatment with (non-selective) c-MET inhibitors cabozantinib and crizotinib (87). A recent *in vitro* study by Charan et al. demonstrated overexpression of HGF in ES xenograft tumors and cell lines, compared to control tissues. The overexpression of HGF was related to an oncogenic p53 isoform, and the authors showed that treatment with a neutralizing anti-HGF antibody could synergize with GD2-targeted CAR-T cell therapy to inhibit ES xenograft growth and increase survival in a murine model (88).

### Glioma

Gliomas are the most common pediatric CNS tumors, which after leukemia are responsible for the most cancer deaths in this population (89). Few effective chemotherapeutic options exist for treatment, especially of higher-grade or non-resectable lesions, and outcomes are frequently quite poor. As these cancers are also common in the adult population, much of the literature is comprised of studies of adult tumors.

Early studies of human primary brain tumor samples have shown increased expression of c-MET and/or HGF in higher grade gliomas, such as glioblastoma multiforme (GBM) (90–94). Microscopy analysis of tumor sections, as well as *in vitro* and *in vivo* experiments with anti-HGF agents, have suggested that both autocrine and paracrine signaling of HGF occurs in gliomas (90, 91, 93, 95–97). Similar to its effects in sarcomas, stimulation with HGF increased glioma cell proliferation, motility, and invasion (90, 91, 94). Treatment with anti-HGF agents, such as anti-HGF snRNA/ribozymes (95), anti-HGF antibodies (96, 98), or competitive inhibitors of HGF (99) decreased the growth of *in vivo* xenograft glioma tumors by decreasing cell proliferation and increasing apoptosis (and through effects on angiogenesis which will be addressed separately). Notably, Kim et al. showed in a murine model that glioma xenografts which express c-MET without co-secreting HGF are completely resistant to treatment with an anti-HGF mAb. However, the authors indicated that these results do not necessarily preclude successful therapy in humans, as any native murine HGF in this model (in other words, paracrine HGF) would not have been targeted by the anti-HGF mAb (98). Martens et al. found that an anti-c-MET antibody was effective against GBM xenografts co-expressing HGF and c-MET, while xenografts expressing c-MET but not secreting (human) HGF were resistant to therapy (97).

Studies comparing HGF/c-MET expression in pathological specimens to clinical outcomes have indicated that greater presence of HGF/c-MET is associated with poorer prognosis and outcomes (though this data is largely from adult patients) (100–103). Interestingly, Kong et al. found that a greater percentage of c-MET overexpressing tumors had aggressive radiographic findings on initial MR imaging (invasive or multifocal lesions) compared to c-MET-negative (or low-expressing) tumors (100).

Tumor neovascularization and angiogenesis play a significant role in glial cancers, particularly in aggressive cancers like GBM. The HGF/c-MET pathway influences angiogenesis both directly and indirectly [reviewed in (104)], and treatment with anti-HGF/c-MET agents can reduce tumor microvessel density (95, 97–99). Studies of HGF/c-MET distribution in human primary brain tumors showed that expression was not limited to glial cancer cells, but was also found in supporting tumor microvasculature (91–93). *In vitro* stimulation with HGF promoted chemotactic migration and increased DNA synthesis in neuro microvascular endothelial cells, suggesting a direct pro-angiogenic role for HGF (94, 105).

Probing the role of HGF as an *indirect* mediator of angiogenesis, Moriyama et al. demonstrated that treatment of glioma cell lines with HGF increased VEGF mRNA expression and protein secretion in a dose-dependent fashion (106). However, Schmidt et al. quantified the levels of VEGF, HGF, and bFGF in extracts from human gliomas, and found that levels of both VEGF and HGF were increased in higher grade gliomas, and were *independent* predictors of microvessel density (107). Using both GBM cell lines and primary cultures, Eckerich et al. found that exposure to hypoxia caused an increase in *MET* transcription and c-MET expression. This effect was linked to hypoxia-inducible-factor 1-alpha (HIF-1a) (the *MET* promoter is known to have HIF-1 binding sites), and was more pronounced in cell lines that had relatively low basal c-MET expression (108). Martens et al. tested the effects of a one-armed anti-c-MET antibody (OA-5D5) on the *in vivo* growth of GBM xenografts, and interestingly found that treatment with OA-5D5 decreased intratumoural microvessel density, despite OA-5D5 being unable to bind murine c-MET. The authors suggested that in this model of GBM, anti-c-MET therapy had an indirect anti-angiogenic effect, potentially by decreasing proteolysis within the tumor extracellular matrix, thereby inhibiting neovascular spread (97).

Cancer stem cells are increasingly recognized as key drivers of therapy resistance and tumor progression, particularly in high-grade cancers such as GBM. Studies of GBM-derived cells and neurospheres have identified an association between HGF/c-MET signaling and “stemness” in GBM [reviewed in (109)]. c-MET signaling within neurospheres (which recapitulate the heterogeneity of a GBM tumor more accurately than single cell cultures) was associated with expression of other identifiers of “stemness,” and treatment with HGF sustained clonogenic potential and expression of reprogramming transcription factors (110). However, rather than homogeneously expressing c-MET, subpopulations within c-MET-positive neurospheres of c-MET high- and low-expressing cells were identified, with the high-expressing subpopulations having greater “stem”-like characteristics, including greater clonogenicity and capacity for multipotential differentiation, and enhanced *in vivo* tumorigenicity (111). Similarly, a study of freshly isolated patient-derived GBM cells showed that within a sorted population of c-MET-positive cells, those with high c-MET expression were more clonogenic, and more tumorigenic in an orthotopic *in vivo* model, than those with lower c-MET expression (112).

A recent study by Qin et al. found that *de novo* glioma formation could be instigated by injecting human HGF and c-MET cDNA in combination with siRNA against TRp53 into the lateral ventricle of neonatal mice. These results suggest that increased HGF/c-MET signaling, on a oncogenic background of p53 attenuation, could be sufficient to transform healthy neural stem cells into malignant glioma stem cells (113).

The HGF/c-MET pathway has been shown to contribute to anti-cancer therapy resistance in human gliomas. Indeed, irradiation of glioma stem cells has been shown to induce c-MET upregulation (112), while *in vitro* studies have shown that pre-treating GBM cells with HGF reduced the cytotoxic effect of DNA damaging agents (114). Furthermore, treatment of GBM xenografts with chimeric U1/ribozymes targeting HGF and c-MET mRNA sensitized tumors to gamma radiation, and greatly improved tumor regression and cure rate (115).

Much of the literature discussing the role of HGF/c-MET in glioma is based upon studies of adult primary tumors, or cell lines derived from adult tumors. However, owing to distinct genetic differences, these findings might not be directly applicable to the pediatric population (116). A recent review of the molecular landscape of pediatric gliomas suggests that in fact pediatric tumors may be a better target for many precision therapies, due to their relative paucity of diverse genetic drivers compared to adult tumors, which tend to be more genetically heterogeneous (117). Wu et al. found amplifications of the *MET* gene in 6% (7/112) of pediatric high-grade gliomas analyzed (118). Bender et al. analyzed the genetic profiles of pediatric GBMs, and found that up to 10% of tumors harbored gene fusions involving *MET*–of all the fusion genes identified, *MET* was the single gene most frequently involved. Notably, all of the GBM samples found to have a *MET* fusion were also found to have impaired cell cycle regulation. Indeed, the authors found that cell lines transduced with a *MET* fusion gene were only tumorigenic on the background of a *TP53* mutation, or deletion of *CDKN2A/CDKN2B*, suggesting that while dysregulation of the c-MET pathway plays a role in tumorigenesis, it alone is not sufficient to drive tumor growth (119).

### Medulloblastoma

Medulloblastoma (MB) is the most common brain malignancy in pediatrics. Due to the risk of leptomeningeal dissemination, current therapy of MB generally includes surgical resection, chemotherapy and radiation of the neuraxis–an aggressive treatment approach which can result in significant morbidity including endocrinological and neurocognitive deficits. MB develops from neural progenitor cells in the posterior fossa, most commonly in the cerebellum—the HGF/c-MET pathway is crucial for normal cerebellar development (120), and dysregulation of this pathway has been implicated as a driver of MB.

An early study of the genetic drivers of MB found single copy gains of *MET* in 38.5% of tumor samples analyzed (121). c-MET and HGF were found to be expressed in tumor specimens and MB cell lines, and levels of c-MET mRNA correlated with poor clinical outcomes. Moreover, it was shown that HGF stimulation of MB cells *in vitro* induced proliferation, anchorage-independent growth, and protection from chemotherapy-induced cell death. Overexpression of HGF *in vivo* led to increased growth of xenografts, with unfavorable histologic phenotypes (122). Small molecule inhibition of c-MET *in vitro* in MB cell lines led to decreased proliferation, motility, and anchorage-independent growth (123), and treatment with an oral c-MET inhibitor (crizotinib) *in vivo* inhibited growth of subcutaneous MB tumor xenografts (124).

Work done to investigate other molecules and pathways involved in the tumorigenesis of MB found that HGF induced expression of c-Myc (an oncoprotein whose presence can indicate poor prognosis in MB), and inhibition or overexpression of c-Myc in MB modulated the effects of HGF on cell cycle progression and proliferation (125). Additionally, epigenome screening identified serine protease inhibitor kunitz-type 2 (SPINT2–an inhibitor of HGF activation) as a potential tumor suppressor gene in MB. A majority of primary MB tumor samples showed decreased SPINT2 expression, while stable re-expression of SPINT2 in MB cell lines resulted in decreased MB cell proliferation, anchorage-independent growth, and longer overall survival of mice implanted with intercerebellar tumor xenografts (126). Investigation into the relationship between the serine/threonine protein kinase MAP4K4 and c-MET found that in certain MB cell lines, blocking MAP4KR signaling affected c-MET signaling (promoted endocytosis and recycling of c-MET), suggesting a potential combination therapeutic target for a subset of MB (127).

MB can be classified into molecular subgroups: wingless/integrated (WNT), sonic Hedgehog (SHH), and groups 3 and 4. Genetic analysis has revealed an association between increased c-MET expression and the SHH subgroup of MB, though increased c-MET expression is also seen in a subset of tumors classified in groups 3 and 4 (128–130). Higher levels of phosphorylated c-MET were correlated with tumor recurrence and poor prognosis in the SHH subgroup of MB (129). Binning et al. found that activation of SHH in murine models induced formation of MB, while overexpression of HGF on the background of activated SHH resulted in an increased frequency of MB formation. Treatment with a mAb against HGF in this model improved overall survival, though the inhibition of tumor growth was incomplete, leading the authors to hypothesize that dual inhibition of both SHH and HGF would be more effective (131). A further study by the same group interestingly revealed that the combination therapy with SHH and HGF inhibitors did not improve survival (and even reduced survival in one combination therapy) compared to monotherapy (132). Separately, small molecule inhibition of c-MET with foretinib has been shown to be effective against subcutaneous and orthotopic xenografts of disseminated SHH subgroup MB, as well as a transgenic mouse model of metastatic SHH MB (129).

### Neuroblastoma

Neuroblastoma (NB) is a malignancy derived from primitive cells of the sympathetic nervous system and is the most common extracranial pediatric solid tumor (133). The clinical behavior and prognosis of NB can range broadly—from tumors which spontaneously regress, to aggressive metastatic tumors causing death.

Hecht et al. provided the first evidence of a role for the HGF/c-MET pathway in NB. They found variable expression of HGF and c-MET across established NB cell lines, with little basal activation of c-MET. However, stimulation of NB cells with HGF promoted invasion and expression of proteolytic extracellular matrix (ECM)-degrading factors. Pre-stimulation of NB cells with HGF in a model of NB in chick embryos (using the chorioallantoic membrane as an epithelial barrier to investigate invasive growth) led to the increased formation of well vascularized tumors (134). A further study by the same group found that expression of the neurotrophin receptor TrkB (a hallmark of invasive and metastatic NB) led to upregulation of HGF and c-MET (and an increase in phosphorylated c-MET, suggesting autocrine stimulation). TrkB-expressing cells were more invasive than the parental cells. A series of stimulation experiments revealed that while anti-BDNF (the stimulating ligand for TrkB) treatment could reduce c-MET phosphorylation, anti-HGF treatment did not similarly affect phosphorylation of TrkB–but did reduce phosphorylation of c-MET (which could be partially rescued by BDNF stimulation). These results suggest that the relationship between TrkB and c-MET is driven at the top by stimulation through TrkB. Analysis of tumor specimens from advanced disease found increased expression of TrkB in a subset of samples, all of which had increased expression of HGF and c-MET (135). Ren et al. investigated the relationship between c-MET and another prognostic marker in NB, macrophage migration inhibitory factor (MIF). When expression of MIF was reduced in an NB cell line, c-MET mRNA levels decreased. Decreased MIF expression led to a less aggressive NB phenotype *in vitro* (less invasive, less proliferative, and more apoptotic), and decreased tumor growth and metastasis *in vivo* (136).

Plasma and serum samples from patients with NB were found to have an overall increased concentration of HGF compared to plasma and serum from healthy controls. Notably, HGF concentration was higher in patients with later stage NB and was associated with negative markers of NB prognosis (137). qPCR analysis of c-MET mRNA in NB tumor samples found a correlation between increased c-MET expression and later clinical stage (138). Yan et al. examined the genomic and protein expression of c-MET in NB clinical samples, and across the 54 samples analyzed they identified one example of *MET* gene amplification, and only one example of increased c-MET expression by IHC. The rest of the samples analyzed did not show any c-MET overexpression, though the authors did identify some alternatively spliced *MET* isoforms (139). Scorsone et al. looked at a small panel of NB patient samples and found detectable c-MET expression in 38% (3/8) of tumor samples (140). Similarly, established NB cell lines have shown variable (often minimal to no) c-MET expression (134, 140, 141).

*In vitro* and pre-clinical studies have suggested the potential therapeutic benefit of small molecule inhibition of c-MET in NB (138, 140). Cabozantinib (a small molecule inhibitor of c-MET and VEGFR among other RTKs) was compared to selective anti-VEGFR inhibition in models of NB, and while anti-VEGFR therapy alone led to increased metastasis, cabozantinib decreased metastatic spread to visceral organs (though not to bones). The study was limited, however, by the use of different animal models and cell lines for the study of metastatic spread after VEGFR inhibition or cabozantinib treatment, and thus a direct comparison of the two therapies is not possible (142).

### Wilms tumor (nephroblastoma)

Wilms tumor (WT), also known as nephroblastoma, is the most common renal malignancy in children. It is an embryonal malignancy and is thought to derive from aberrant differentiation and development in early nephrogenesis. HGF/c-MET autocrine and paracrine signaling has been shown to play a part in early kidney development (143), and so a role for this pathway in WT tumorigenesis has been posited. The data on a role (if any) for HGF/c-MET in WT is, however, limited. A screen of solid tumors for mutations in the *MET* gene revealed no mutations in 52 samples of WT (144). In 2002, Alami et al. showed that in a relatively small subset of WT samples, expressions of c-MET and HGF were raised compared to normal kidney tissue, and that in a majority of samples higher levels of c-MET were correlated with a marker of proliferation (145). In contrast, a later study by Vuononvirta et al. reported tissue microarray analysis of a larger cohort of WT and showed that only 8% (15/193) and 14% (25/179) of non-anaplastic tumors expressed HGF or c-MET, respectively, and did not find an association with markers of proliferation. They found greater HGF expression in nephrogenic rests (precursor lesions to WT) compared to WT, suggesting a loss of this signaling pathway with development of malignancy (146). To date the role of HGF/c-MET in WT remains unclear.

### Hepatoblastoma

While rare overall, hepatoblastoma (HB) is the most common pediatric liver malignancy (with some geographical differences), particularly in younger children and infants. HB is thought to arise from early hepatic progenitor cells, and owing to their pluripotent nature, histological subtypes of HB include both epithelial and mixed epithelial-mesenchymal variants (147). As previously discussed, HGF/c-MET plays a crucial role in normal liver development and regeneration (14, 20, 22, 23), and so it is perhaps unsurprising that this pathway has been implicated in the pathogenesis of HB.

In pediatric patients, liver regeneration after partial hepatectomy is particularly robust and rapid. In some patients undergoing partial hepatectomy for untreated HB, rapid recurrence of liver tumors and lung metastases is seen post-operatively, coinciding with the time period of maximal liver regeneration. HGF is a crucial growth factor involved in liver regeneration. Von Schweinitz et al. found that sera from HB pediatric patients had significantly elevated HGF concentrations post-hepatectomy compared to healthy controls (with no statistically significant difference in serum HGF concentrations pre-treatment compared to healthy controls). HGF was shown to be produced by stromal cells in co-culture with HB cells, suggesting a paracrine mechanism of HGF stimulation, while analysis of tumor sections revealed high expression of c-MET by epithelial tumor cells. In a subset of patients, HB tumors were compared to normal liver tissue (from the same patient, *n* = 5) and c-MET mRNA levels were found to be upregulated in HB tumor samples compared to healthy liver tissue (148). The authors of this early paper also showed that *in vitro*, HGF at physiologic concentrations (matching clinical data of serum HGF concentrations post-hepatectomy) increased proliferation of established HB cell lines, while higher (non-physiologic) concentrations of HGF had the opposite effect (148). However, a more recent study with the same established HB lines and similar concentrations of HGF found that HGF stimulation did not increase viability or proliferation of HB cells, but rather increased HB cell survival only in the face of stressors like serum-starvation, or treatment with chemotherapy, an effect that was mediated through the PI3K/AKT downstream signaling pathway (149).

Aberrant accumulation of β-catenin in the cytoplasm and nucleus can be seen in a large proportion of HB (150), and is likely involved in HB tumorigenesis [reviewed in (147)]. While activation of β-catenin occurs classically through the Wnt pathway, Wnt-independent and HGF-induced nuclear translocation of β-catenin has been shown to occur as a result of a subcellular interaction of β-catenin and c-MET (151). Clinical data suggests significant but variable rates of mutation/deletion in the gene for β-catenin (*CTNNB1*) affecting β-catenin accumulation in HB. Purcell et al. studied clinical samples of HB, and while 87% (85/98) of tumors showed aberrant β-catenin accumulation, the frequency of *CTNNB1* mutations in their patient cohort was substantially lower [at most 20% (20/98) of samples], suggesting an alternative mechanism for β-catenin accumulation. Significant levels of c-MET activated β-catenin [Y654-β-catenin, (152)] were found in the cytoplasm and nucleus of clinical HB samples with wild-type *CTNNB1*, suggesting that aberrant accumulation β-catenin in these cases was due to HGF/c-MET signaling, a mechanism which was validated in a wild-type *CTNNB1* HB cell line after HGF stimulation (153). Recently, Matsumoto et al. found that overexpression of c-MET in addition to overexpression of constitutively active β-catenin and YAP (Yes-associated protein) *in vivo* led to the formation of murine liver tumors similar to human HB and expressing HB tumor markers (154).

An early study examined the role of various genetic alterations on patient outcomes with HB, and found that clinical outcomes after multimodal therapy were not correlated with *MET* RNA expression levels, though it should be noted that only 23 patient samples had *MET* levels quantified (155).

### Childhood hepatocellular carcinoma

Hepatocellular carcinoma (HCC) is becoming increasingly prevalent worldwide, although it remains relatively rare in childhood (with geographic differences in incidence). In the vast majority of adult HCC, an etiology related to chronic liver injury/inflammation (alcoholic/non-alcoholic fatty liver disease, viral liver infection etc.) is identified, and patients often present in the context of liver cirrhosis. In children, however, HCC more often develops on the background of a non-cirrhotic liver, and is often considered a separate entity (156, 157).

In 1999, Park et al. sequenced the TK domain of the *MET* gene in primary liver tumors, from both pediatric and adult patients. Interestingly, they found that 30% (3/10) of childhood HCC samples had missense mutations in the TK domain of the *MET* gene, while no *MET* mutations were found across 16 adult HCC tumors. The authors suggest that potentially activating mutations of the *MET* kinase domain could contribute to the tumorigenesis of childhood HCC, and account for the relatively accelerated timeline compared to adult HCC (which often occurs in the context of longstanding liver inflammation and cirrhosis) (158).

The HGF/c-MET pathway has been explored in adult HCC and is reviewed in (159, 160). Briefly, c-MET has been shown to be overexpressed in a proportion of HCC samples compared to normal liver tissue, and has been linked to advanced disease or poor prognosis (161–165). However, the proportion of tumors overexpressing c-MET varies considerably between studies (160). Pre-clinical studies have shown both pro- and anti-tumorigenic roles for the HGF/c-MET pathway in HCC (166–169), and analysis of clinical samples suggests an overall decreased expression of HGF in adult HCC (160).

Wang et al. showed that transgenic mice with overexpression of c-MET by hepatocytes developed HCC, and that this tumorigenesis was likely due to c-MET activation by cell attachment rather than by HGF stimulation. Furthermore, when expression of c-MET was inhibited, established HCC tumors regressed dramatically, suggesting a role for c-MET in both the development and maintenance of HCC (170). Tao et al. identified a subset of human HCC cases (9–12.5% of analyzed samples) containing both c-MET overexpression and mutation of the β-catenin gene (*CTNNB1*), and showed that overexpression of c-MET on the background of mutant β-catenin led to the formation of HCC in transgenic mice (171).

### Thyroid malignancies

A study by Ramirez et al. examined the implications of c-MET expression with respect to recurrence and metastasis risk in children and young adults with papillary thyroid carcinoma (PTC). Immunohistochemical staining intensities of benign and malignant thyroid lesions were analyzed with respect to clinical outcome. The specimens examined included PTC, follicular thyroid carcinomas (FTC), medullary thyroid carcinomas (MTC), as well as specimens from patients with benign thyroid conditions and normal thyroid tissues. This study found that enhanced expression of c-MET and HGF/SF may be associated with an increased risk for metastasis and tumor recurrence in this patient population (172).

Table 1 broadly summarizes the known effects of HGF/c-MET signaling in the range of pediatric tumor types reviewed here.

**TABLE 1:** Effects of the HGF/c-MET pathway in pediatric tumors.

Effects of HGF/c-MET signaling	Tumor types in which effect has been observed
Increased cell invasion/metastasis	RMS, OS, glioma, MB, NB
Increased tumor neovascularization	Glioma, NB
Increased resistance to chemotherapy/radiotherapy	RMS, glioma, MB, HB
Decreased cell differentiation	RMS, OS, glioma
Increased cell proliferation	Glioma, MB, HB

## Hepatocyte growth factor/mesenchymal epithelial transition factor pathway as a therapeutic target

Recently, a number of agents that target the HGF/c-MET pathway have been developed and evaluated in preclinical and clinical studies of various cancers. These agents include small molecule inhibitors for c-MET kinase activity, as well as anti-c-MET and anti-HGF antibodies. Currently available small molecule inhibitors include bozitinib, cabozantinib, crizotinib, MSC2156119J, MK-2461, AMG-337, capmatinib, tepotinib, amuvatinib, elzovantinib, savolitinib, glumetinib, and golvatinib (173–176). These molecules have distinct characteristics with respect to their ability to interact with specific residues and regions in the active c-MET molecule. For example, bozitinib (PLB-1001) is a highly selective ATP-competitive c-MET inhibitor with blood-brain barrier permeability allowing for its potential utility in secondary GBM, where a significant proportion of patients carry *METex14* alterations which are associated with a poor prognosis (177). Additionally, a number of monoclonal antibodies against c-MET and HGF, as well as competitive analogs of HGF have been developed to target the HGF/c-MET pathway in tumors (178). Compared to small molecule tyrosine kinase inhibitors (TKIs) that often interact with multiple cellular growth regulatory kinases, antibody-based therapeutics inhibit components of the HGF/c-MET pathway with greater specificity, interrupting the ligand binding process. For this reason, these therapeutics are more likely to be effective against tumors with c-MET overexpression, and not restricted to those with oncogenic molecular alterations (175).

Therapeutics targeting the HGF/c-MET pathway tested in clinical trials thus far include c-MET TKIs (selective for only c-MET or targeting additional RTKs), anti-c-MET antibodies, and anti-HGF antibodies. Fu et al. recently published a comprehensive review of clinical trials of HGF/c-MET targeted therapies in adult cancers, which span these three categories of therapeutics (179). Pediatric trials of HGF/c-MET targeted therapies have been more limited, and have thus far only tested selective and non-selective c-MET tyrosine kinase inhibitors (TKIs). Table 2 summarizes the current landscape of completed and recruiting clinical trials of HGF/c-MET targeted therapies with pediatric eligibility and recruitment.

**TABLE 2:** Active, recruiting, or completed clinical trials of HGF/c-MET targeting agents that include pediatric patients.

Drug name	Known target(s)	Trial number	Description	Status/References
AMG-337	c-MET	NCT03132155	Phase 2 study in clear cell sarcoma containing EWSR1-ATF1 gene fusion	Active, not recruiting
Tivantinib	c-MET	NCT01725191	Phase 1 study in pediatric solid tumors	Completed (183)
		NCT00557609	Phase 2 study in MiT tumors	Completed (184)
Savolitinib	c-MET	NCT03598244	Phase 1 study in pediatric CNS tumors with *MET* aberrations	Recruiting
Crizotinib	c-MET, ALK, ROS1	NCT01644773	Phase 1 study of the combination of crizotinib and dasatinib in pediatric DIPG and HGG	Completed (195, 196)
		NCT00939770	Phase 1/2 study in pediatric solid tumors and ALCL	Completed (190, 191, 197)
		NCT01606878	Phase 1 study of crizotinib in combination with conventional chemotherapy in pediatric solid tumors or ALCL	Completed (198)
		NCT01979536	Phase 2 study of brentuximab vedotin or crizotinib in combination with chemotherapy in pediatric patients with ALCL	Active, not recruiting
		NCT02034981	Phase 2 study in diverse tumors with an alteration in *ALK*, *MET*, or *ROS1*	Active, not recruiting (192)
		NCT01121588	Phase 1 study of crizotinib in tumors with aberrations of *ALK* (note, mutations in *MET* but not *ALK* are in exclusion criteria)	Active, not recruiting (199)
		NCT01744652	Phase 1 study of dasatinib in combination with crizotinib in advanced solid malignancies (or lymphoma)	Completed (200)
		NCT04283669	Phase 2 study in children and adults with neurofibromatosis type 2 and progressive vestibular schwannomas	Active, not recruiting
		NCT01524926	Phase 2 study in advanced tumors with alterations of *ALK* and/or c-MET pathways	Active, not recruiting (193, 194, 201, 202)
		NCT02638428	Phase 2 study of various targeted therapies in pediatric tumors based on genetic sequencing	Recruiting
		NCT02693535	Phase 2 study of various targeted therapies in a range of tumors based on genomic variants or known drug targets	Recruiting
Cabozantinib	c-MET, VEGFR2, AXL, RET, KIT, FLT3	NCT03611595	Phase I study of cabozantinib in combination with 13-*cis*-Retinoic Acid in pediatric solid tumors	Recruiting
		NCT01709435	Phase 1 study in pediatric solid tumors (incl. CNS tumors)	Completed (187)
		NCT02867592	Phase 2 study in refractory sarcomas, WT, and other rare tumors	Active, not recruiting (188)
		NCT04661852	Phase 1 study of cabozantinib with topotecan and cyclophosphamide in ES or OS	Active, not recruiting
		NCT02101736	Phase II study in patients with neurofibromatosis type 1 and plexiform neurofibromas	Active, not recruiting (203)
		NCT02243605	Phase 2 study in OS and ES	Active, not recruiting (186)

Studies with inclusion criteria <18 years of age that did not end up recruiting pediatric patients in reported results are excluded.

Some studies of crizotinib are explicitly targeting ALK not c-MET but have been included here for completeness.

### Tivantinib

Tivantinib (ARQ 197), a staurosporine derivative, was identified as a highly selective inhibitor of the c-MET tyrosine kinase, capable of inhibiting c-MET phosphorylation and its downstream effects (180). Tivantinib has also demonstrated significant cytotoxic activity independent of c-MET inhibition, and a mechanism of action involving microtubule dynamics has been posited (181, 182). The uncertainty surrounding tivantinib’s main mechanism of action has important implications for clinical trial design and appropriate biomarker selection.

In the pediatric population, tivantinib has been investigated as a c-MET inhibitor in multiple cancer types. A Children’s Oncology Group phase I trial in pediatric patients with relapsed/refractory solid tumors investigated oral dosing and pharmacokinetics. While oral tivantinib was well tolerated, there was marked pharmacokinetic variability among patients, and no objective responses were seen. Tumor samples (solid tumors including CNS tumors) were analyzed for c-MET expression by IHC, and the majority of samples [81% (26/32)] were found to have undetectable levels of c-MET (183).

Tivantinib has also been evaluated in a phase II trial in adolescent and adult patients (median age of 25) with advanced microphthalmic transcription factor (MITF)-associated tumors (including alveolar soft part sarcoma, translocation-associated renal cell carcinoma, and clear cell sarcoma). The best response in the study was a partial response (PR) in a patient with clear cell sarcoma. Baseline tumor expression of c-MET was evaluated by IHC for 19 patients (out of *n* = 27). The majority of tumors evaluated were either highly or focally positive for c-MET [74% (14/19)], but no correlation with c-MET staining and best response was observed. Of note, the one patient with clear cell sarcoma who had a PR was negative for c-MET staining (184).

Thus far, the clinical activity of tivantinib in pediatric patients has been limited, and the specificity of this agent as a targeted anti-c-MET therapeutic is uncertain.

### Cabozantinib

Cabozantinib is a TKI with multiple targets including c-MET, VEGFR2, RET, KIT, AXL, and FLT3. Cabozantinib has both direct anti-tumor activity and anti-angiogenic effects (185).

A recently reported multi-center phase II clinical trial (the CABONE trial) investigated the effects of cabozantinib in ES and OS. Though the trial was primarily conducted in adult patients (the median age of OS patients was 34), the results showed that in patients with OS, treatment with cabozantinib gave rise to a 12% objective response rate and 33% of patients had 6 months of non-progression (meeting the study’s primary efficacy criterion). Moreover, a biomarker analysis indicated that an increased soluble (plasma) c-MET level at baseline was correlated with improved progression free survival, suggesting that this subpopulation of OS patients with high c-MET expression was responding to the anti-c-MET therapy (186). In a heavily pre-treated ES patient population, an objective response rate of 26% was seen (all PRs), and the primary efficacy criterion was met. However, biomarker analysis did not reveal any associations between serum c-MET and survival outcomes, and as cabozantinib also targets VEGFR2, it is not clear if in these cases the treatment benefit derived primarily from targeting c-MET activity or the anti-angiogenic effects of targeting VEGFR2 (186).

The Children’s Oncology Group has also investigated cabozantinib in relapsed or refractory solid tumors. After a phase I trial found that cabozantinib led to some PRs or stable disease (SD) (187), a phase II trial was conducted to determine activity. The trial was divided into six cohorts: OS (*n* = 29), ES, RMS, non-RMS soft tissue sarcoma, WT (all *n* = 13), and other rare tumors (*n* = 23). Overall, in OS 10/29 patients (34%) had disease control after 4 months (2 PR and 8 SD), while no partial or complete responses were seen in patients with EWS, RMS, or WT (though some PRs were seen in other rare tumors). The study showed potential for cabozantinib in treatment of OS, but limited efficacy in other sarcomas or WT (188).

A recent single-institution case series reported on the use of cabozantinib in four pediatric patients with relapsed NB. All four patients had extended disease control after treatment with cabozantinib for at least 6 months, while two patients had complete responses (>12 months) and remained in clinical remission at the time of publication (189). It is, however, unclear whether this benefit derived from targeting c-MET or rather targeting of VEGFR (or other RTKs sensitive to inhibition by cabozantinib).

### Crizotinib

Crizotinib is another non-selective TKI targeting c-MET, though many of the clinical studies of crizotinib have focused primarily on its role targeting anaplastic lymphoma kinase (ALK). In the adult population, crizotinib is approved for treatment of NSCLC with altered *ALK*.

In pediatric populations, crizotinib has been investigated in a variety of solid tumors and anti-tumor activity has been correlated with the presence of *ALK* aberrations (190, 191). Crizotinib is approved for treatment of relapsed or refractory anaplastic large cell lymphoma with altered *ALK*.

From the perspective of targeting c-MET, a number of phase I and II trials which included children and adolescents in their eligibility criteria have examined the role of crizotinib in tumors with *MET* alterations.

The AcSé program in France supported a multi-tumor phase II trial wherein 107 pediatric tumors were analyzed for alterations in the molecular targets of crizotinib (*ALK*, *MET*, and *ROS-1*). From this cohort, 11 patients with genetic alterations were enrolled and received crizotinib (results from only eight patients were available at the time of abstract publication). The tumor analysis found one *MET* translocation, and three instances of *MET* amplification. Notably the single patient with a *MET* translocation (glioma) had progressive disease with crizotinib therapy (192).

The European Organization for Research and Treatment of Cancer (EORTC) “CREATE” trial was designed as a multi-center multi-tumor trial to evaluate crizotinib in a range of cancer types known to harbor alterations in the targets of crizotinib (*ALK*, *MET*, and *ROS-1*) (193). The trial was open to patients aged 16 and older, and though the patient cohort consisted primarily of adults, the trial contained cohorts of patients with cancers more commonly seen in children, adolescents, and young adults, and so is discussed here.

In advanced ARMS (positive for MET fusion protein), crizotinib (as a single agent) did not have meaningful clinical activity, with just one patient achieving a PR as the best outcome. As only eight patients out of 13 were evaluable for the primary end-point due to the aggressive course of their disease, the authors suggest that targeted therapeutics like crizotinib might be better trialed in aggressive cancers as an earlier treatment (in combination with standard chemotherapy). Given the lack of disease response to anti-c-MET therapy, the authors postulate that c-MET activation is likely not a key driver of disease progression at this advanced stage in ARMS, but may play a role in earlier differentiation (194).

The combination of crizotinib and another TKI, dasatinib, was tested in a Phase I trial in pediatric patients with advanced high-grade glioma or diffuse intrinsic pontine gliomas. Unfortunately, the combination was poorly tolerated, and little anti-tumor activity was appreciated. Notably, only 13% (2/16) of cases available for molecular analysis revealed *MET* amplification (195).

## Discussion

Clearly, the HGF/c-MET pathway contributes to a wide variety of pediatric malignancies. Given its crucial role in development, proliferation, cell motility, and regeneration this is perhaps unsurprising, as aberrations in these cellular functions are commonly identified as drivers of oncogenesis and metastatic spread. There is ample evidence in the literature reviewed above to suggest that targeting the HGF/c-MET pathway could be an effective anti-cancer strategy across a range of tumor types. This promise has not, however, been fully borne out in the limited number of pediatric clinical trials that have been completed to date. Overall, the HGF/c- MET pathway is an exciting target and has shown early promise in cancers such as osteosarcoma. However, in many of the tumor types reviewed here, the principal effects of dysregulated HGF/c-MET signaling have largely been shown to be an increase in invasive/metastatic behavior, resistance to chemo- or radiotherapy, and a promotion of poorly differentiated “stem-like” cells, as opposed to driving cell proliferation and growth. This is likely reflected in the relative paucity of objective clinical responses seen in pediatric clinical trials to date. With the potential exception of tumors containing growth-sustaining genetic alterations in *MET*, treatment of tumors with anti-HGF/c-MET agents will likely be most impactful in preventing disease progression or relapse after treatment or resection, and when used in combination with cytotoxic therapies. Multiple pre-clinical studies reviewed above suggest that the HGF/c-MET pathway is important for protecting cancer cells from insult, and so it follows that anti-HGF/c-MET therapy could be effective as an adjuvant to other cytotoxic therapies. One of the key hurdles in cancer therapy is overcoming drug resistance developed within heavily pre-treated refractory tumors and targeting the HGF/c-MET pathway could be a promising tool for addressing this.

It is also important to acknowledge that, while invaluable for pre-clinical research and drug development, *in vitro* models generally do not reflect the diversity seen across tumors in patients. Many of the studies reviewed above included analysis of HGF/c-MET expression in patient samples, though there existed considerable variability in the methods for quantification, and the “normal” controls used between reports. Biomarker analysis for appropriate patient selection is crucial for any targeted therapy to achieve its predicted potential from pre-clinical data, although in early stages of development it is important to include open Phase I/II studies as to not exclude any groups who might potentially benefit from the therapy for reasons not yet fully elucidated. There are currently multiple ongoing or recruiting trials of anti-c-MET agents including pediatric patients, hence it is expected that additional key data will become available regarding the safety and efficacy of these agents for childhood cancers in the near future.

## Author contributions

MG and AN contributed to the review of the literature and writing of the manuscript. Both authors contributed to the article and approved the submitted version.

## References

[1] Stoker M Gherardi E Perryman M Gray J. Scatter factor is a fibroblast-derived modulator of epithelial cell mobility. *Nature.* (1987) 327:239–42. 10.1038/327239a0 2952888

[2] Gherardi E Gray J Stoker M Perryman M Furlong R. Purification of scatter factor, a fibroblast-derived basic protein that modulates epithelial interactions and movement. *Proc Natl Acad Sci U.S.A.* (1989) 86:5844–8. 10.1073/pnas.86.15.5844 2527367 PMC297727

[3] Birchmeier C Birchmeier W Gherardi E Vande Woude GF. Met, metastasis, motility and more. *Nat Rev Mol Cell Biol.* (2003) 4:915–25. 10.1038/nrm1261 14685170

[4] Gherardi E Birchmeier W Birchmeier C Woude G. Vande. Targeting MET in cancer: rationale and progress. *Nat Rev Cancer.* (2012) 12:89–103. 10.1038/nrc3205 22270953

[5] Koch JP Aebersold DM Zimmer Y Medová M. MET targeting: time for a rematch. *Oncogene.* (2020) 39:2845–62. 10.1038/s41388-020-1193-8 32034310

[6] Park M Dean M Kaul K Braun MJ Gonda MA Vande Woude G. Sequence of MET protooncogene cDNA has features characteristic of the tyrosine kinase family of growth-factor receptors. *Proc Natl Acad Sci U.S.A.* (1987) 84:6379–83. 10.1073/pnas.84.18.6379 2819873 PMC299079

[7] Cooper CS Park M Blair DG Tainsky MA Huebner K Croce CM Molecular cloning of a new transforming gene from a chemically transformed human cell line. *Nature.* (1984) 311:29–33. 10.1038/311029a0 6590967

[8] Park M Dean M Cooper CS Schmidt M O’Brien SJ Blair DG Mechanism of met oncogene activation. *Cell.* (1986) 45:895–904. 10.1016/0092-8674(86)90564-7 2423252

[9] Weidner KM Arakaki N Hartmann G Vandekerckhove J Weingart S Rieder H Evidence for the identity of human scatter factor and human hepatocyte growth factor. *Proc Natl Acad Sci U.S.A.* (1991) 88:7001–5. 10.1073/pnas.88.16.7001 1831266 PMC52221

[10] Bottaro DP Rubin JS Faletto DL Chan AML Kmiecik TE Vande Woude GF Identification of the hepatocyte growth factor receptor as the c-met proto-oncogene product. *Science.* (1991) 251:802–4. 10.1126/science.1846706 1846706

[11] Trusolino L Bertotti A Comoglio PM. MET signalling: principles and functions in development, organ regeneration and cancer. *Nat Rev Mol Cell Biol.* (2010) 11:834–48. 10.1038/nrm3012 21102609

[12] Weidner KM Behrens J Vandekerckhove J Birchmeier W. Scatter factor: molecular characteristics and effect on the invasiveness of epithelial cells. *J Cell Biol.* (1990) 111:2097–108. 10.1083/jcb.111.5.2097 2146276 PMC2116316

[13] Zhu H Naujokas MA Park M. Receptor chimeras indicate that the met tyrosine kinase mediates the motility and morphogenic responses of hepatocyte growth/scatter factor. *Cell Growth Dif.* (1994) 5:359–66. 8043510

[14] Schmidt C Bladt F Goedecke S Brlnkmann V Zschiesche W Sharpet M Scatter factor/hepatocyte essential for liver development. *Nature.* (1995) 373:699–702. 10.1038/373699a0 7854452

[15] Bladt F Riethmacher D Stefan I Aguzzi A Birchmeier C. Essential role for the c-mef receptor in the migration of myogenlc precursor cells into the limb bud. *Nature.* (1995) 376:768–71. 10.1038/376768a0 7651534

[16] Uehara Y Minowa O Mori C Shiota K Kuno J Noda T Placental defect and embryonic lethality in mice lacking hepatocyte growth factor/scatter factor. *Nature.* (1995) 373:702–5. 10.1038/373702a0 7854453

[17] Matsumori A Furukawa Y Hashimoto T Ono K Shioi T Okada M Increased circulating hepatocyte growth factor in the early stage of acute myocardial infarction. *Biochem Biophys Res Commun.* (1996) 221:391–5. 10.1006/bbrc.1996.0606 8619866

[18] Nakamura T Mizuno S Matsumoto K Sawa Y. Myocardial protection from ischemia/reperfusion injury by endogenous and exogenous HGF. *J Clin Invest.* (2000) 106:1511–9. 10.1172/JCI10226 11120758 PMC387252

[19] Igawa T Matsumoto K Kanda S Saito Y Nakamura T. Hepatocyte growth factor may function as a renotropic factor for regeneration in rats with acute renal injury. *Am J Physiol Ren Fluid Electrolyte Physiol.* (1993) 265:61–9. 10.1152/ajprenal.1993.265.1.F61 8342615

[20] Borowiak M Garratt AN Wüstefeld T Strehle M Trautwein C Birchmeier C. Met provides essential signals for liver regeneration. *Proc Natl Acad Sci U.S.A.* (2004) 101:10608–13. 10.1073/pnas.0403412101 15249655 PMC490025

[21] Yanagita K Matsumoto K Sekiguchi K Ishibashi H Niho Y Nakamura T. Hepatocyte growth factor may act as a pulmotrophic factor on lung regeneration after acute lung injury. *J Biol Chem.* (1993) 268:21212–7. 10.1016/S0021-9258(19)36912-1 8407957

[22] Huh CG Factor VM Sánchez A Uchida K Conner EA Thorgeirsson SS. Hepatocyte growth factor/c-met signaling pathway is required for efficient liver regeneration and repair. *Proc Natl Acad Sci U.S.A.* (2004) 101:4477–82. 10.1073/pnas.0306068101 15070743 PMC384772

[23] Ishikawa T Factor VM Marquardt JU Raggi C Seo D Kitade M Hepatocyte growth factor/c-met signaling is required for stem-cell-mediated liver regeneration in mice. *Hepatology.* (2012) 55:1215–26. 10.1002/hep.24796 22095660 PMC3299882

[24] Chmielowiec J Borowiak M Morkel M Stradal T Munz B Werner S c-Met is essential for wound healing in the skin. *J Cell Biol.* (2007) 177:151–62. 10.1083/jcb.200701086 17403932 PMC2064119

[25] Zhou D Tan RJ Lin L Zhou L Liu Y. Activation of hepatocyte growth factor receptor, c-met, in renal tubules is required for renoprotection after acute kidney injury. *Kidney Int.* (2013) 84:509–20. 10.1038/ki.2013.102 23715119 PMC3758808

[26] Ponzetto C Bardelli A Zhen Z Maina F dalla Zonca P Giordano S A multifunctional docking site mediates signaling and transformation by the hepatocyte growth factor/scatter factor receptor family. *Cell.* (1994) 77:261–71. 10.1016/0092-8674(94)90318-2 7513258

[27] Weidner KM Di Cesare S Sachs M Brinkmann V Behrens J. Interaction between Gab1 and the c-Met receptor tyrosine kinase is responsible for epithelial morphogenesis. *Nature.* (1996) 384:173–6. 10.1038/384173a0 8906793

[28] Fixman Fournier TM Kamikura DM Naujokas MA Park M. Pathways downstream of Shc and Grb2 are required for cell transformation by the Tpr-Met oncoprotein. *J Biol Chem.* (1996) 271:13116–22. 10.1074/jbc.271.22.13116 8662733

[29] Boccaccio C Andò M Tamagnone L Bardelli A Michieli P Battistini C Induction of epithelial tubules by growth factor HGF depends on the STAT pathway. *Nature.* (1998) 391:285–8. 10.1038/34657 9440692

[30] Maroun CR Naujokas MA Holgado-Madruga M Wong AJ Park M. The tyrosine phosphatase SHP-2 is required for sustained activation of extracellular signal-regulated kinase and epithelial morphogenesis downstream from the met receptor tyrosine kinase. *Mol Cell Biol.* (2000) 20:8513–25. 10.1128/MCB.20.22.8513-8525.2000 11046147 PMC102157

[31] Schaeper U Gehring NH Fuchs KP Sachs M Kempkes B Birchmeier W. Coupling of Gab1 to c-Met. Grb2, and Shp2 mediates biological responses. *J Cell Biol.* (2000) 149:1419–32. 10.1083/jcb.149.7.1419 10871282 PMC2175135

[32] Lai AZ Abella JV Park M. Crosstalk in Met receptor oncogenesis. *Trends Cell Biol.* (2009) 19:542–51. 10.1016/j.tcb.2009.07.002 19758803

[33] Ishibe S Karihaloo A Ma H Zhang J Marlier A Mitobe M Met and the epidermal growth factor receptor act cooperatively to regulate final nephron number and maintain collecting duct morphology. *Development.* (2009) 136:337–45. 10.1242/dev.024463 19103805 PMC2862758

[34] Turke AB Zejnullahu K Wu YL Song Y Dias-Santagata D Lifshits E Preexistence and clonal selection of MET amplification in EGFR mutant NSCLC. *Cancer Cell.* (2010) 17:77–88. 10.1016/j.ccr.2009.11.022 20129249 PMC2980857

[35] Engelman JA Zejnullahu K Mitsudomi T Song Y Hyland C Joon OP MET amplification leads to gefitinib resistance in lung cancer by activating ERBB3 signaling. *Science.* (2007) 316:1039–43. 10.1126/science.1141478 17463250

[36] Jeffers M Taylor GA Weidner KM Omura S Vande Woude GF. Degradation of the met tyrosine kinase receptor by the ubiquitin-proteasome pathway. *Mol Cell Biol.* (1997) 17:799–808. 10.1128/MCB.17.2.799 9001234 PMC231806

[37] Peschard P Fournier TM Lamorte L Naujokas MA Band H Langdon WY Mutation of the c-Cbl TKB domain binding site on the Met receptor tyrosine kinase converts it into a transforming protein. *Mol Cell.* (2001) 8:995–1004. 10.1016/S1097-2765(01)00378-1 11741535

[38] Hammond DE Urbé S Vande Woude GF Clague MJ. Down-regulation of MET, the receptor for hepatocyte growth factor. *Oncogene.* (2001) 20:2761–70. 10.1038/sj.onc.1204475 11420688

[39] Petrelli A Gilestro GF Lanzardo S Comoglio PM Migone N Giordano S. The endophilin-CIN85-Cbl complex mediates ligand-dependent downregulation of c-Met. *Nature.* (2002) 416:187–90. 10.1038/416187a 11894096

[40] Gandino L Longati P Medico E Prat M Comoglio PM. Phosphorylation of serine 985 negatively regulates the hepatocyte growth factor receptor kinase. *J Biol Chem.* (1994) 269:1815–20. 10.1016/S0021-9258(17)42099-0 8294430

[41] Hashigasako A Machide M Nakamura T Matsumoto K Nakamura T. Bi-directional regulation of Ser-985 phosphorylation of c-Met via protein kinase C and protein phosphatase 2A involves c-Met activation and cellular responsiveness to hepatocyte growth factor. *J Biol Chem.* (2004) 279:26445–52. 10.1074/jbc.M314254200 15075332

[42] Foveau B Ancot F Leroy C Petrelli A Reiss K Vingtdeux V Down-regulation of the met receptor tyrosine kinase by presenilin-dependent regulated intramembrane proteolysis. *Mol Biol Cell.* (2009) 20:2495–2250. 10.1091/mbc.e08-09-0969 19297528 PMC2675628

[43] Palka HL Park M Tonks NK. Hepatocyte growth factor receptor tyrosine kinase met is a substrate of the receptor protein-tyrosine phosphatase DEP-1. *J Biol Chem.* (2003) 278:5728–35. 10.1074/jbc.M210656200 12475979

[44] Machide M Hashigasako A Matsumoto K Nakamura T. Contact inhibition of hepatocyte growth regulated by functional association of the c-Met/hepatocyte growth factor receptor and LAR protein-tyrosine phosphatase. *J Biol Chem.* (2006) 281:8765–72. 10.1074/jbc.M512298200 16415345

[45] Sangwan V Paliouras GN Abella JV Dubé N Monast A Tremblay ML Regulation of the met receptor-tyrosine kinase by the protein-tyrosine phosphatase 1B and T-cell phosphatase. *J Biol Chem.* (2008) 283:34374–83. 10.1074/jbc.M805916200 18819921 PMC2662243

[46] Comoglio PM Trusolino L Boccaccio C. Known and novel roles of the MET oncogene in cancer: a coherent approach to targeted therapy. *Nat Rev Cancer.* (2018) 18:341–58. 10.1038/s41568-018-0002-y 29674709

[47] Liang TJ Reid AE Xavier R Cardiff RD Wang TC. Transgenic expression of tpr-met oncogene leads to development of mammary hyperplasia and tumors. *J Clin Invest.* (1996) 97:2872–7. 10.1172/JCI118744 8675700 PMC507382

[48] Soman NR Correa P Ruiz BA Wogan GN. The TPR-MET oncogenic rearrangement is present and expressed in human gastric carcinoma and precursor lesions. *Proc Natl Acad Sci U.S.A.* (1991) 88:4892–6. 10.1073/pnas.88.11.4892 2052572 PMC51773

[49] Skapek SX Ferrari A Gupta A Lupo PJ Butler E Barr FG Rhabdomyosarcoma. *Nat Rev Dis Prim.* (2019) 5:1. 10.1038/s41572-018-0051-2 30617281 PMC7456566

[50] Parham DM Barr FG. Classification of rhabdomyosarcoma and its molecular basis. *Adv Anat Pathol.* (2013) 20:387. 10.1097/PAP.0b013e3182a92d0d 24113309 PMC6637949

[51] Breneman JC Lyden E Pappo AS Link MP Anderson JR Parham DM Prognostic factors and clinical outcomes in children and adolescents with metastatic rhabdomyosarcoma-a report from the intergroup rhabdomyosarcoma study IV. *J Clin Oncol.* (2003) 21:78–84. 10.1200/JCO.2003.06.129 12506174

[52] Oberlin O Rey A Lyden E Bisogno G Stevens MCG Meyer WH Prognostic factors in metastatic rhabdomyosarcomas: results of a pooled analysis from United States and European cooperative groups. *J Clin Oncol.* (2008) 26:2384–9. 10.1200/JCO.2007.14.7207 18467730 PMC4558625

[53] Bennicelli JL Advani S Schäfer BW Barr FG. PAX3 and PAX7 exhibit conserved cis-acting transcription repression domains and utilize a common gain of function mechanism in alveolar rhabdomyosarcoma. *Oncogene.* (1999) 18:4348–56. 10.1038/sj.onc.1202812 10439042

[54] Skapek SX Anderson J Barr FG Bridge JA Gastier-Foster JM Parham DM PAX-FOXO1 fusion status drives unfavorable outcome for children with rhabdomyosarcoma: a children’s oncology group report. *Pediatr Blood Cancer.* (2013) 60:1411–7. 10.1002/pbc.24532 23526739 PMC4646073

[55] Relaix F Polimeni M Rocancourt D Ponzetto C Schäfer BW Buckingham M. The transcriptional activator PAX3-FKHR rescues the defects of Pax3 mutant mice but induces a myogenic gain-of-function phenotype with ligand-independent activation of Met signaling in vivo. *Genes Dev.* (2003) 17:2950–65. 10.1101/gad.281203 14665670 PMC289153

[56] Sharp R Recio JA Jhappan C Otsuka T Liu S Yu Y Synergism between INK4a/ARF inactivation and aberrant HGF/SF signaling in rhabdomyosarcomagenesis. *Nat Med.* (2002) 8:1276–80. 10.1038/nm787 12368906

[57] Ginsberg JP Davis RJ Bennicelli JL Nauta LE Barr FG. Up-regulation of MET but not neural cell adhesion molecule expression by the PAX3-FKHR fusion protein in alveolar rhabdomyosarcoma. *Cancer Res.* (1998) 58:3542–6. 10.1097/00043426-199807000-00084 9721857

[58] Taulli R Scuoppo C Bersani F Accornero P Forni PE Miretti S Validation of met as a therapeutic target in alveolar and embryonal rhabdomyosarcoma riccardo. *Cancer Res.* (2006) 66:4742–9. 10.1016/j.critrevonc.2018.08.007 16651427

[59] Jankowski K Kucia M Wysoczynski M Reca R Zhao D Trzyna E Both hepatocyte growth factor (HGF) and stromal-derived factor-1 regulate the metastatic behavior of human rhabdomyosarcoma cells, but only HGF enhances their resistance to radiochemotherapy. *Cancer Res.* (2003) 63:7926–35. 14633723

[60] Lukasiewicz E Miekus K Kijowski J Drabik G Wilusz M Bobis-Wozowicz S Inhibition of rhabdomyosarcoma’s metastatic behavior through downregulation of MET receptor signaling. *Folia Histochem Cytobiol.* (2009) 47:485–9. 10.2478/v10042-009-0108-x 20164036

[61] Diomedi-Camassei F McDowell HP De loris MA Uccini S Altavista P Raschellà G Clinical significance of CXC chemokine receptor-4 and c-Met in childhood rhabdomyosarcoma. *Clin Cancer Res.* (2008) 14:4119–27. 10.1158/1078-0432.CCR-07-4446 18593989

[62] Miekus K Lukasiewicz E Jarocha D Sekula M Drabik G Majka M. The decreased metastatic potential of rhabdomyosarcoma cells obtained through MET receptor downregulation and the induction of differentiation. *Cell Death Dis.* (2013) 4:1–10. 10.1038/cddis.2012.199 23328666 PMC3563987

[63] Anastasi S Giordano S Sthandier O Gambarotta G Maione R Comoglio P A natural hepatocyte growth factor/scatter factor autocrine loop in myoblast cells and the effect of the constitutive met kinase activation on myogenic differentiation. *J Cell Biol.* (1997) 137:1057–68. 10.1083/jcb.137.5.1057 9166406 PMC2136220

[64] Skrzypek K Kusienicka A Szewczyk B Adamus T Lukasiewicz E Miekus K Constitutive activation of MET signaling impairs myogenic differentiation of rhabdomyosarcoma and promotes its development and progression. *Oncotarget.* (2015) 6:31378–98. 10.18632/oncotarget.5145 26384300 PMC4741613

[65] Yan D Dong Xda E Chen X Wang L Lu C Wang J MicroRNA-1/206 targets c-met and inhibits rhabdomyosarcoma development. *J Biol Chem.* (2009) 284:29596–604. 10.1074/jbc.M109.020511 19710019 PMC2785592

[66] Taulli R Bersani F Foglizzo V Linari A Vigna E Ladanyi M The muscle-specific microRNA miR-206 blocks human rhabdomyosarcoma growth in xenotransplanted mice by promoting myogenic differentiation. *J Clin Invest.* (2009) 119:2366–78. 10.1172/JCI38075 19620785 PMC2719932

[67] Chen Y Takita J Mizuguchi M Tanaka K Ida K Koh K Mutation and expression analyses of the MET and CDKN2A in rhabdomyosarcoma with emphasis on MET overexpression. *Genes Chromosom Cancer.* (2007) 46:348–58. 10.1002/gcc.20416 17243166

[68] Du J Wang Y Meng L Liu Y Pang Y Cui W c-MET expression potentially contributes to the poor prognosis of rhabdomyosarcoma. *Int J Clin Exp Pathol.* (2018) 11:4083–92. 31949799 PMC6962817

[69] Anderson ME. Update on survival in osteosarcoma. *Orthop Clin North Am.* (2016) 47:283–92. 10.1016/j.ocl.2015.08.022 26614941

[70] Ferracini R Di Renzo MF Scotlandi K Baldini N Olivero M Lollini P The MET/HGF receptor is over-expressed in human osteosarcomas and is activated by either a paracrine or autocrine circuit. *Oncogene.* (1995) 10:739–49. 7862451

[71] Scotlandi K Baldini N Oliviero M Di Renzo MF Martano M Serra M Expression of met/hepatocyte growth factor receptor gene and malignant behavior of musculoskeletal tumors. *Am J Pathol.* (1996) 149:1209–19. 8863670 PMC1865197

[72] Oda Y Naka T Takeshita M Iwamoto Y Tsuneyoshi M. Comparison of histological changes and changes in nm23 and c-MET expression between primary and metastatic sites in osteosarcoma: a clinicopathologic and immunohistochemical study. *Hum Pathol.* (2000) 31:709–16. 10.1053/hupa.2000.8230 10872665

[73] Naka T Iwamoto Y Shinohara N Ushijima M Chuman H Tsuneyoshi M. Expression of c-met proto-oncogene product (c-MET) in benign and malignant bone tumors. *Mod Pathol.* (1997) 10:832–8. 9267827

[74] Coltella N Manara MC Cerisano V Trusolino L Di Renzo MF Scotlandi K Role of the MET/HGF receptor in proliferation and invasive behavior of osteosarcoma. *FASEB J.* (2003) 17:1162–4. 10.1096/fj.02-0576fje 12709413

[75] Hassan SE Bekarev M Kim MY Lin J Piperdi S Gorlick R Cell surface receptor expression patterns in osteosarcoma. *Cancer.* (2012) 118:740–9. 10.1002/cncr.26339 21751203

[76] Patanè S Avnet S Coltella N Costa B Sponza S Olivero M MET overexpression turns human primary osteoblasts into osteosarcomas. *Cancer Res.* (2006) 66:4750–7. 10.1158/0008-5472.CAN-05-4422 16651428

[77] Dani N Olivero M Mareschi K Van Duist MM Miretti S Cuvertino S The MET oncogene transforms human primary bone-derived cells into osteosarcomas by targeting committed osteo-progenitors. *J Bone Miner Res.* (2012) 27:1322–34. 10.1002/jbmr.1578 22367914

[78] Duan Z Choy E Harmon D Liu X Susa M Mankin H MicroRNA-199a-3p is downregulated in human osteosarcoma and regulates cell proliferation and migration. *Mol Cancer Ther.* (2011) 10:1337–45. 10.1158/1535-7163.MCT-11-0096 21666078 PMC3711153

[79] Yan K Gao J Yang T Ma Q Qiu X Fan Q MicroRNA-34a inhibits the proliferation and metastasis of osteosarcoma cells both in vitro and in vivo. *PLoS One.* (2012) 7:e33778. 10.1371/journal.pone.0033778 22457788 PMC3310405

[80] Niu G Li B Sun J Sun L. miR-454 is down-regulated in osteosarcomas and suppresses cell proliferation and invasion by directly targeting c-Met. *Cell Prolif.* (2015) 48:348–55. 10.1111/cpr.12187 25880599 PMC6496886

[81] Li X Sun X Wu J Li Z. MicroRNA-613 suppresses proliferation, migration and invasion of osteosarcoma by targeting c-MET. *Am J Cancer Res.* (2016) 6:2869–79. 28042506 PMC5199760

[82] Georges S Calleja LR Jacques C Lavaud M Moukengue B Lecanda F Loss of miR-198 and -206 during primary tumor progression enables metastatic dissemination in human osteosarcoma. *Oncotarget.* (2018) 9:35726–41. 10.18632/oncotarget.26284 30515265 PMC6254661

[83] Xie W Xiao J Wang T Zhang D Li Z. MicroRNA-876-5p inhibits cell proliferation, migration and invasion by targeting c-Met in osteosarcoma. *J Cell Mol Med.* (2019) 23:3293–301. 10.1111/jcmm.14217 30773847 PMC6484334

[84] Li Q Lu C Wang J Gao M Gao W. MicroRNA-449b-5p suppresses proliferation, migration, and invasian of osteosarcoma by targeting c-Met. *Med Sci Monit.* (2019) 25:6236–43. 10.12659/MSM.918454 31425497 PMC6713030

[85] Sampson ER Martin BA Morris AE Xie C Schwarz EM O’Keefe RJ The orally bioavailable met inhibitor PF-2341066 inhibits osteosarcoma growth and osteolysis/matrix production in a xenograft model. *J Bone Miner Res.* (2011) 26:1283–94. 10.1002/jbmr.336 21308771

[86] Fioramonti M Fausti V Pantano F Iuliani M Ribelli G Lotti F Cabozantinib affects osteosarcoma growth through a direct effect on tumor cells and modifications in bone microenvironment. *Sci Rep.* (2018) 8:1–11. 10.1038/s41598-018-22469-5 29520051 PMC5843583

[87] Fleuren EDG Roeffen MHS Leenders WP Flucke UE Vlenterie M Schreuder HW Expression and clinical relevance of MET and ALK in Ewing sarcomas. *Int J Cancer.* (2013) 133:427–36. 10.1002/ijc.28047 23335077

[88] Charan M Dravid P Cam M Audino A Gross AC Arnold MA GD2-directed CAR-T cells in combination with HGF-targeted neutralizing antibody (AMG102) prevent primary tumor growth and metastasis in Ewing sarcoma. *Int J Cancer.* (2020) 146:3184–95. 10.1002/ijc.32743 31621900 PMC7440656

[89] Ostrom QT Gittleman H Fulop J Liu M Blanda R Kromer C CBTRUS statistical report: primary brain and central nervous system tumors diagnosed in the United States in 2008-2012. *Neuro Oncol.* (2015) 17:iv1–62. 10.1093/neuonc/nov189 26511214 PMC4623240

[90] Rosen EM Laterra J Joseph A Jin L Fuchs A Way D Scatter factor expression and regulation in human glial tumors. *Int J Cancer.* (1996) 67:248–55. 10.1002/(SICI)1097-0215(19960717)67:2<248::AID-IJC16>3.0.CO;2-7 8760595

[91] Koochekpour S Jeffers M Rulong S Taylor G Klineberg E Hudson EA Met and hepatocyte growth factor/scatter factor expression in human gliomas. *Cancer Res.* (1997) 57:5391–8. 9393765

[92] Nabeshima K Shimao Y Sato S Kataoka H Moriyama T Kawano H Expression of c-Met correlates with grade of malignancy in human astrocytic tumours: an immunohistochemical study. *Histopathology.* (1997) 31:436–43. 10.1046/j.1365-2559.1997.3010889.x 9416484

[93] Kunkel P Müller S Schirmacher P Stavrou D Fillbrandt R Westphal M Expression and localization of scatter factor/hepatocyte growth factor in human astrocytomas. *Neuro Oncol.* (2001) 3:82–8. 10.1093/neuonc/3.2.82 11296484 PMC1920608

[94] Lamszus K Schmidt NO Jin L Laterra J Zagzag D Way D Scatter factor promotes motility of human glioma and neuromicrovascular endothelial cells. *Int J Cancer.* (1998) 75:19–28. 10.1002/(SICI)1097-0215(19980105)75:1<19::AID-IJC4>3.0.CO;2-4 9426685

[95] Abounader R Lal B Luddy C Koe G Davidson B Rosen EM In vivo targeting of SF/HGF and c-met expression via U1snRNA/ribozymes inhibits glioma growth and angiogenesis and promotes apoptosis. *FASEB J.* (2002) 16:108–10. 10.1096/fj.01-0421fje 11729097

[96] Cao B Su Y Oskarsson M Zhao P Kort EJ Fisher RJ Neutralizing monoclonal antibodies to hepatocyte growth factor/scatter factor (HGF/SF) display antitumor activity in animal models. *Proc Natl Acad Sci U.S.A.* (2001) 98:7443–8. 10.1073/pnas.131200498 11416216 PMC34688

[97] Martens T Schmidt NO Eckerich C Filibrandt R Merchant M Schwall R A novel one-armed anti-c-Met antibody inhibits glioblastoma growth in vivo. *Clin Cancer Res.* (2006) 12:6144–52. 10.1158/1078-0432.CCR-05-1418 17062691

[98] Kim KJ Wang L Su YC Gillespie GY Salhotra A Lal B Systemic anti-hepatocyte growth factor monoclonal antibody therapy induces the regression of intracranial glioma xenografts. *Clin Cancer Res.* (2006) 12:1292–8. 10.1158/1078-0432.CCR-05-1793 16489086

[99] Brockmann MA Papadimitriou A Brandt M Fillbrandt R Westphal M Lamszus K. Inhibition of intracerebral glioblastoma growth by local treatment with the scatter factor/hepatocyte growth factor-antagonist NK4. *Clin Cancer Res.* (2003) 9:4578–85. 14555533

[100] Kong DS Song SY Kim DH Kyeung MJ Yoo JS Jong SK Prognostic significance of c-Met expression in glioblastomas. *Cancer.* (2009) 115:140–8. 10.1002/cncr.23972 18973197

[101] Pierscianek D Kim YH Motomura K Mittelbronn M Paulus W Brokinkel B MET gain in diffuse astrocytomas is associated with poorer outcome. *Brain Pathol.* (2013) 23:13–8. 10.1111/j.1750-3639.2012.00609.x 22672415 PMC8028935

[102] Olmez OF Cubukcu E Evrensel T Kurt M Avci N Tolunay S The immunohistochemical expression of c-Met is an independent predictor of survival in patients with glioblastoma multiforme. *Clin Transl Oncol.* (2014) 16:173–7. 10.1007/s12094-013-1059-4 23740136

[103] Petterson SA Dahlrot RH Hermansen SK K A. Munthe S Gundesen MT Wohlleben H High levels of c-Met is associated with poor prognosis in glioblastoma. *J Neurooncol.* (2015) 122:517–27. 10.1007/s11060-015-1723-3 25800004

[104] Abounader R Laterra J. Scatter factor/hepatocyte growth factor in brain tumor growth and angiogenesis. *Neuro Oncol.* (2005) 7:436–51. 10.1215/S1152851705000050 16212809 PMC1871724

[105] Brockmann MA Ulbricht U Grüner K Fillbrandt R Westphal M Lamszus K Glioblastoma and cerebral microvascular endothelial cell migration in response to tumor-associated growth factors. *Neurosurgery.* (2003) 52:1391–9. 10.1227/01.NEU.0000064806.87785.AB 12762884

[106] Moriyama T Kataoka H Hamasuna R Yokogami K Uehara H Kawano H Up-regulation of vascular endothelial growth factor induced by hepatocyte growth factor/scatter factor stimulation in human glioma cells. *Biochem Biophys Res Commun.* (1998) 249:73–7. 10.1006/bbrc.1998.9078 9705834

[107] Schmidt NO Westphal M Hagel C Ergün S Stavrou D Rosen EM Levels of vascular endothelial growth factor, hepatocyte growth factor/scatter factor and basic fibroblast growth factor in human gliomas and their relation to angiogenesis. *Int J Cancer.* (1999) 84:10–8. 10.1002/(SICI)1097-0215(19990219)84:1<10::AID-IJC3>3.0.CO;2-L 9988225

[108] Eckerich C Zapf S Fillbrandt R Loges S Westphal M Lamszus K. Hypoxia can induce c-Met expression in glioma cells and enhance SF/HGF-induced cell migration. *Int J Cancer.* (2007) 121:276–83. 10.1002/ijc.22679 17372907

[109] Boccaccio C Comoglio PM. The MET oncogene in glioblastoma stem cells: implications as a diagnostic marker and a therapeutic target. *Cancer Res.* (2013) 73:3193–9. 10.1158/0008-5472.CAN-12-4039 23695554

[110] Li Y Li A Glas M Lal B Ying M Sang Y c-Met signaling induces a reprogramming network and supports the glioblastoma stem-like phenotype. *Proc Natl Acad Sci U.S.A.* (2011) 108:9951–6. 10.1073/pnas.1016912108 21628563 PMC3116406

[111] De Bacco F Casanova E Medico E Pellegatta S Orzan F Albano R The MET oncogene is a functional marker of a glioblastoma stem cell subtype. *Cancer Res.* (2012) 72:4537–50. 10.1158/0008-5472.CAN-11-3490 22738909

[112] Joo KM Jin J Kim E Kim KH Kim Y Kang BG MET signaling regulates glioblastoma stem cells. *Cancer Res.* (2012) 72:3828–38. 10.1158/0008-5472.CAN-11-3760 22617325

[113] Qin Y Musket A Kou J Preiszner J Tschida BR Qin A Overexpression of HGF/MET axis along with p53 inhibition induces de novo glioma formation in mice. *Neuro-Oncology Adv.* (2020) 2:1–12. 10.1093/noajnl/vdaa067 32642717 PMC7332240

[114] Bowers DC Fan S Walter KA Abounader R Williams JA Rosen EM Scatter factor/hepatocyte growth factor protects against cytotoxic death in human glioblastoma via phosphatidylinositol 3-kinase- and AKT-dependent pathways. *Cancer Res.* (2000) 60:4277–83. 10945642

[115] Lal B Xia S Abounader R Laterra J. Targeting the c-Met pathway potentiates glioblastoma responses to γ-radiation. *Clin Cancer Res.* (2005) 11:4479–86. 10.1158/1078-0432.CCR-05-0166 15958633

[116] Mackay A Burford A Carvalho D Izquierdo E Fazal-Salom J Taylor KR Integrated molecular meta-analysis of 1,000 pediatric high-grade and diffuse intrinsic pontine glioma. *Cancer Cell.* (2017) 32:520.e–37.e. 10.1016/j.ccell.2017.08.017 28966033 PMC5637314

[117] Miklja Z Pasternak A Stallard S Nicolaides T Kline-Nunnally C Cole B Molecular profiling and targeted therapy in pediatric gliomas: review and consensus recommendations. *Neuro Oncol.* (2019) 21:968–80. 10.1093/neuonc/noz022 30805642 PMC6682212

[118] Wu G Diaz AK Paugh BS Rankin SL Ju B Li Y The genomic landscape of diffuse intrinsic pontine glioma and pediatric non-brainstem high-grade glioma. *Nat Genet.* (2014) 46:444–50. 10.1038/ng.2938 24705251 PMC4056452

[119] Bender S Gronych J Warnatz HJ Hutter B Gröbner S Ryzhova M Recurrent MET fusion genes represent a drug target in pediatric glioblastoma. *Nat Med.* (2016) 22:1314–20. 10.1038/nm.4204 27748748

[120] Ieraci A Forni PE Ponzetto C. Viable hypomorphic signaling mutant of the met receptor reveals a role for hepatocyte growth factor in postnatal cerebellar development. *Proc Natl Acad Sci U.S.A.* (2002) 99:15200–5. 10.1073/pnas.222362099 12397180 PMC137567

[121] Tong CYK Hui ABY Yin XL Pang JCS Zhu XL Poon WS Detection of oncogene amplifications in medulloblastomas by comparative genomic hybridization and array-based comparative genomic hybridization. *J Neurosurg.* (2004) 100:187–93. 10.3171/ped.2004.100.2.0187 14758948

[122] Li Y Lal B Kwon S Fan X Saldanha U Reznik TE The scatter factor/hepatocyte growth factor: c-Met pathway in human embryonal central nervous system tumor malignancy. *Cancer Res.* (2005) 65:9355–62. 10.1158/0008-5472.CAN-05-1946 16230398

[123] Kongkham PN Onvani S Smith CA Rutka JT. Inhibition of the met receptor tyrosine kinase as a novel therapeutic strategy in medulloblastoma. *Transl Oncol.* (2010) 3:336–43. 10.1593/tlo.10121 21151472 PMC3000458

[124] Guessous F Yang Y Johnson E Marcinkiewicz L Smith M Zhang Y Cooperation between c-Met and focal adhesion kinase family members in medulloblastoma and implications for therapy. *Mol Cancer Ther.* (2012) 11:288–97. 10.1158/1535-7163.MCT-11-0490 22188814 PMC3277676

[125] Li Y Guessous F Johnson EB Eberhart CG Li XN Shu Q Functional and molecular interactions between the HGF/c-Met pathway and c-Myc in large-cell medulloblastoma. *Lab Investig.* (2008) 88:98–111. 10.1038/labinvest.3700702 18059365

[126] Kongkham PN Northcott PA Ra YS Nakahara Y Mainprize TG Croul SE An epigenetic genome-wide screen identifies SPINT2 as a novel tumor suppressor gene in pediatric medulloblastoma. *Cancer Res.* (2008) 68:9945–53. 10.1158/0008-5472.CAN-08-2169 19047176

[127] Tripolitsioti D Kumar KS Neve A Migliavacca J Capdeville C Rushing EJ MAP4K4 controlled integrin β1 activation and c-Met endocytosis are associated with invasive behavior of medulloblastoma cells. *Oncotarget.* (2018) 9:23220–36. 10.18632/oncotarget.25294 29796184 PMC5955425

[128] Onvani S Terakawa Y Smith C Northcott P Taylor M Rutka J. Molecular genetic analysis of the hepatocyte growth factor/MET signaling pathway in pediatric medulloblastoma. *Genes Chromosom Cancer.* (2012) 57:675–88. 10.1002/gcc.21954 22447520

[129] Faria CC Golbourn BJ Dubuc AM Remke M Diaz RJ Agnihotri S Foretinib is effective therapy for metastatic sonic hedgehog medulloblastoma. *Cancer Res.* (2015) 75:134–46. 10.1158/0008-5472.CAN-13-3629 25391241

[130] Santhana Kumar K Tripolitsioti D Ma M Grählert J Egli KB Fiaschetti G The Ser/Thr kinase MAP4K4 drives c-Met-induced motility and invasiveness in a cell-based model of SHH medulloblastoma. *Springerplus.* (2015) 4:19. 10.1186/s40064-015-0784-2 25625039 PMC4302160

[131] Binning MJ Niazi T Pedone CA Lal B Eberhart CG Kim KJ Hepatocyte growth factor and Sonic hedgehog expression in cerebellar neural progenitor cells costimulate medulloblastoma initiation and growth. *Cancer Res.* (2008) 68:7838–45. 10.1158/0008-5472.CAN-08-1899 18829539 PMC2638505

[132] Coon V Laukert T Pedone CA Laterra J Kim KJ Fults DW. Molecular therapy targeting sonic hedgehog and hepatocyte growth factor signaling in a mouse model of medulloblastoma. *Mol Cancer Ther.* (2010) 9:2627–36. 10.1158/1535-7163.MCT-10-0486 20807782 PMC2937075

[133] Van Arendonk KJ Chung DH. Neuroblastoma: tumor biology and its implications for staging and treatment. *Children.* (2019) 6:12. 10.3390/children6010012 30658459 PMC6352222

[134] Hecht M Papoutsi M Tran HD Wilting J Schweigerer L. Hepatocyte growth factor/c-Met signaling promotes the progression of experimental human neuroblastomas. *Cancer Res.* (2004) 64:6109–18. 10.1158/0008-5472.CAN-04-1014 15342394

[135] Hecht M Schulte JH Eggert A Wilting J Schweigerer L. The neurotrophin receptor TrkB cooperates with c-Met in enhancing neuroblastoma invasiveness. *Carcinogenesis.* (2005) 26:2105–15. 10.1093/carcin/bgi192 16051641

[136] Ren Y Chan HM Fan J Xie Y Chen YX Li W Inhibition of tumor growth and metastasis in vitro and in vivo by targeting macrophage migration inhibitory factor in human neuroblastoma. *Oncogene.* (2006) 25:3501–8. 10.1038/sj.onc.1209395 16449971

[137] Sköldenberg EG Larsson A Jakobson Å Hedborg F Kogner P Christofferson RH The angiogenic growth factors HGF and VEGF in serum and plasma from neuroblastoma patients. *Anticancer Res.* (2009) 29:3311–9. 19661350

[138] Crosswell HE Dasgupta A Alvarado CS Watt T Christensen JG De P PHA665752, a small-molecule inhibitor of c-Met, inhibits hepatocyte growth factor-stimulated migration and proliferation of c-Met-positive neuroblastoma cells. *BMC Cancer.* (2009) 9:1–10. 10.1186/1471-2407-9-411 19939254 PMC2790467

[139] Yan B Lim M Zhou L Kuick CH Leong MY Yong KJ Identification of MET genomic amplification, protein expression and alternative splice isoforms in neuroblastomas. *J Clin Pathol.* (2013) 66:985–91. 10.1136/jclinpath-2012-201375 23801497

[140] Scorsone K Zhang L Woodfield SE Hicks J Zage PE. The novel kinase inhibitor EMD1214063 is effective against neuroblastoma. *Invest New Drugs.* (2014) 32:815–24. 10.1007/s10637-014-0107-4 24832869

[141] Zhang L Scorsone K Woodfield SE Zage PE. Sensitivity of neuroblastoma to the novel kinase inhibitor cabozantinib is mediated by ERK inhibition. *Cancer Chemother Pharmacol.* (2015) 76:977–87. 10.1007/s00280-015-2871-z 26407819

[142] Daudigeos-Dubus E Le Dret L Bawa O Opolon P Vievard A Villa I Dual inhibition using cabozantinib overcomes HGF/MET signaling mediated resistance to pan-VEGFR inhibition in orthotopic and metastatic neuroblastoma tumors. *Int J Oncol.* (2017) 50:203–11. 10.3892/ijo.2016.3792 27922668

[143] Woolf AS Kolatsi-Joannou M Hardman P Andermarcher E Moorby C Fine LG Roles of hepatocyte growth factor/scatter factor and the met receptor in the early development of the metanephros. *J Cell Biol.* (1995) 128:171–84. 10.1083/jcb.128.1.171 7822413 PMC2120323

[144] Schmidt L Junker K Nakaigawa N Kinjerski T Weirich G Miller M Novel mutations of the MET proto-oncogene in papillary renal carcinomas. *Oncogene.* (1999) 18:2343–50. 10.1038/sj.onc.1202547 10327054

[145] Alami J Williams BRG Yeger H. Expression and localization of HGF and met in Wilms’ tumours. *J Pathol.* (2002) 196:76–84. 10.1002/path.997 11748645

[146] Vuononvirta R Sebire NJ Messahel B Perusinghe N Reis-Filho JS Pritchard-Jones K Expression of hepatocyte growth factor and its receptor met in wilms’ tumors and nephrogenic rests reflects their roles in kidney development. *Clin Cancer Res.* (2009) 15:2723–30. 10.1158/1078-0432.CCR-08-1898 19318497 PMC2682776

[147] Bell D Ranganathan S Tao J Monga SPS. Novel advances in understanding of molecular pathogenesis of hepatoblastoma: a Wnt/β-Catenin perspective. *Gene Expr.* (2017) 17:141–54. 10.3727/105221616X693639 27938502 PMC5311458

[148] Von Schweinitz D Faundez A Teichmann B Birnbaum T Koch A Hecker H Hepatocyte growth-factor-scatter factor can stimulate post-operative tumor-cell proliferation in childhood hepatoblastoma. *Int J Cancer.* (2000) 85:151–9. 10.1002/(SICI)1097-0215(20000115)85:2<151::AID-IJC1>3.0.CO;2-6 10629070

[149] Grotegut S Kappler R Tarimoradi S Lehembre F Christofori G Von Schweinitz D. Hepatocyte growth factor protects hepatoblastoma cells from chemotherapy-induced apoptosis by AKT activation. *Int J Oncol* (2010) 36:1261–7. 10.3892/ijo_00000610 20372801

[150] Ranganathan S Tan X Monga SPS. B -Catenin and met deregulation in childhood hepatoblastomas. *Pediatr Dev Pathol.* (2005) 8:435–47. 10.1007/s10024-005-0028-5 16211454

[151] Monga SPS Mars WM Pediaditakis P Bell A Mulé K Bowen WC Hepatocyte growth factor induces Wnt-independent nuclear translocation of β-catenin after Met-β-catenin dissociation in hepatocytes. *Cancer Res.* (2002) 62:2064–71. 11929826

[152] Zeng G Apte U Micsenyi A Bell A Monga SPS. Tyrosine residues 654 and 670 in beta-catenin are crucial in regulation of Met-β-catenin interactions. *Cell.* (2006) 312:3620–30. 10.1016/j.yexcr.2006.08.003 16952352 PMC1820835

[153] Purcell R Childs M Maibach R Miles C Turner C Zimmermann A HGF/c-Met related activation of beta-catenin in hepatoblastoma. *J Exp Clin Cancer Res.* (2011) 30:17–9. 10.1186/1756-9966-30-96 21992464 PMC3207961

[154] Matsumoto S Yamamichi T Shinzawa K Kasahara Y Nojima S Kodama T GREB1 induced by Wnt signaling promotes development of hepatoblastoma by suppressing TGFβ signaling. *Nat Commun.* (2019) 10:3882. 10.1038/s41467-019-11533-x 31462641 PMC6713762

[155] von Schweinitz D Kraus JA Albrecht S Koch A Fuchs J Pietsch T. Prognostic impact of molecular genetic alterations in hepatoblastoma. *Med Pediatr Oncol.* (2002) 38:104–8. 10.1002/mpo.1280 11813174

[156] Schmid I von Schweinitz D. Pediatric hepatocellular carcinoma: challenges and solutions. *J Hepatocell Carcinoma.* (2017) 4:15–21. 10.2147/JHC.S94008 28144610 PMC5248979

[157] Weeda VB Aronson DC Verheij J Lamers WH. Is hepatocellular carcinoma the same disease in children and adults? Comparison of histology, molecular background, and treatment in pediatric and adult patients. *Pediatr Blood Cancer.* (2019) 66:1–9. 10.1002/pbc.27475 30259629

[158] Park WS Dong SM Kim SY Na EY Shin MS Pi JH Somatic mutations in the kinase domain of the MET/hepatocyte growth factor receptor gene in childhood hepatocellular carcinomas. *Cancer Res.* (1999) 59:307–10. 9927037

[159] Goyal L Muzumdar MD Zhu AX. Targeting the HGF/c-MET pathway in hepatocellular carcinoma. *Clin Cancer Res.* (2013) 19:2310–8. 10.1158/1078-0432.CCR-12-2791 23388504 PMC4583193

[160] Giordano S Columbano A. Met as a therapeutic target in HCC: facts and hopes. *J Hepatol.* (2014) 60:442–52. 10.1016/j.jhep.2013.09.009 24045150

[161] Suzuki K Hayashi N Yamada Y Yoshihara H Miyamoto Y Ito Y Expression of the c-met protooncogene in human hepatocellular carcinoma. *Hepatology.* (1994) 20:1231–6. 10.1002/hep.1840200520 7927256

[162] Ueki T Fujimoto J Suzuki T Yamamoto H Okamoto E. Expression of hepatocyte growth factor and its receptor, the c-met proto-oncogene, in hepatocellular carcinoma. *Hepatology.* (1997) 25:619–23. 10.1002/hep.510250321 9049208

[163] D’Errico A Fiorentino M Ponzetto A Daikuhara Y Tsubouchi H Brechot C Liver hepatocyte growth factor does not always correlate with hepatocellular proliferation in human liver lesions: its specific receptor c- met does. *Hepatology.* (1996) 24:60–4. 10.1002/hep.510240112 8707284

[164] Wang ZL Liang P Dong BW Yu XL Yu DJ. Prognostic factors and recurrence of small hepatocellular carcinoma after hepatic resection or microwave ablation: a retrospective study. *J Gastrointest Surg.* (2008) 12:327–37. 10.1007/s11605-007-0310-0 17943391

[165] Kondo S Ojima H Tsuda H Hashimoto J Morizane C Ikeda M Clinical impact of c-Met expression and its gene amplification in hepatocellular carcinoma. *Int J Clin Oncol.* (2013) 18:207–13. 10.1007/s10147-011-0361-9 22218908

[166] Shiota G Rhoads DB Wang TC Nakamura T Schmidt EV. Hepatocyte growth factor inhibits growth of hepatocellular carcinoma cells. *Proc Natl Acad Sci U.S.A.* (1992) 89:373–7. 10.1073/pnas.89.1.373 1309612 PMC48239

[167] Horiguchi N Takayama H Toyoda M Otsuka T Fukusato T Merlino G Hepatocyte growth factor promotes hepatocarcinogenesis through c-Met autocrine activation and enhanced angiogenesis in transgenic mice treated with diethylnitrosamine. *Oncogene.* (2002) 21:1791–9. 10.1038/sj.onc.1205248 11896611

[168] Liu ML Mars WM Michalopoulos GK. Hepatocyte growth factor inhibits cell proliferation in vivo of rat hepatocellular carcinomas induced by diethylnitrosamine. *Carcinogenesis.* (1995) 16:841–3. 10.1093/carcin/16.4.841 7728965

[169] Takami T Kaposi-Novak P Uchida K Gomez-Quiroz LE Conner EA Factor VM Loss of hepatocyte growth factor/c-Met signaling pathway accelerates early stages of N-nitrosodiethylamine-induced hepatocarcinogenesis. *Cancer Res.* (2007) 67:9844–51. 10.1158/0008-5472.CAN-07-1905 17942915

[170] Wang R Ferrell LD Faouzi S Maher JJ Michael Bishop J. Activation of the met receptor by cell attachment induces and sustains hepatocellular carcinomas in transgenic mice. *J Cell Biol.* (2001) 153:1023–33. 10.1083/jcb.153.5.1023 11381087 PMC2174327

[171] Tao J Xu E Zhao Y Singh S Xiaolei L Couchy G Modeling a human hcc subset in mice through co- expression of met and point-mutant β-catenin. *Hepatology.* (2016) 64:1587–605. 10.1002/hep.28601 27097116 PMC5073058

[172] Ramirez R Hsu D Patel A Fenton C Dinauer C Tuttle RM Over-expression of hepatocyte growth factor/scatter factor (HGF/SF) and the HGF/SF receptor (cMET) are associated with a high risk of metastasis and recurrence for children and young adults with papillary thyroid carcinoma. *Clin Endocrinol.* (2000) 53:635–44. 10.1046/j.1365-2265.2000.01124.x 11106926

[173] Puccini A Marin-Ramos NI Bergamo F Schirripa M Lonardi S Lenz H-J Safety and tolerability of c-MET inhibitors in cancer. *Drug Saf.* (2019) 42:211–33. 10.1007/s40264-018-0780-x 30649748 PMC7491978

[174] Wang H Rao B Lou J Li J Liu Z Li A The function of the HGF/c-Met axis in hepatocellular carcinoma. *Front Cell Dev Biol.* (2020) 8:1–15. 10.3389/fcell.2020.00055 32117981 PMC7018668

[175] Hong L Zhang J Heymach JV Le X. Current and future treatment options for MET exon 14 skipping alterations in non-small cell lung cancer. *Ther Adv Med Oncol.* (2021) 13:1–16. 10.1177/1758835921992976 33643443 PMC7890719

[176] Goździk-Spychalska J Szyszka-Barth K Spychalski Ł Ramlau K Wójtowicz J Batura-Gabryel H c-MET inhibitors in the treatment of lung cancer. *Curr Treat Options Oncol.* (2014) 15:670–82. 10.1007/s11864-014-0313-5 25266653

[177] Hu H Mu Q Bao Z Chen Y Liu Y Chen J Mutational landscape of secondary glioblastoma guides MET-targeted trial in brain tumor. *Cell.* (2018) 175:1665.e–78.e. 10.1016/j.cell.2018.09.038 30343896

[178] Kim KH Kim H. Progress of antibody-based inhibitors of the HGF-cMET axis in cancer therapy. *Exp Mol Med.* (2017) 49:e307–307. 10.1038/emm.2017.17 28336955 PMC5382561

[179] Fu J Su X Li Z Deng L Liu X Feng X HGF/c-MET pathway in cancer: from molecular characterization to clinical evidence. *Oncogene.* (2021) 40:4625–51. 10.1038/s41388-021-01863-w 34145400

[180] Munshi N Jeay S Li Y Chen CR France DS Ashwell MA ARQ 197, a novel and selective inhibitor of the human c-Met receptor tyrosine kinase with antitumor activity. *Mol Cancer Ther.* (2010) 9:1544–53. 10.1158/1535-7163.MCT-09-1173 20484018

[181] Katayama R Aoyama A Yamori T Qi J Oh-hara T Song Y Cytotoxic activity of tivantinib (ARQ 197) is not due solely to c-MET inhibition. *Cancer Res.* (2013) 73:3087–96. 10.1158/0008-5472.CAN-12-3256 23598276 PMC3759033

[182] Basilico C Pennacchietti S Vigna E Chiriaco C Arena S Bardelli A Tivantinib (ARQ197) displays cytotoxic activity that is independent of its ability to bind MET. *Clin Cancer Res.* (2013) 19:2381–92. 10.1158/1078-0432.CCR-12-3459 23532890

[183] Geller JI Perentesis JP Liu X Minard CG Kudgus RA Reid JM A phase 1 study of the c-Met inhibitor, tivantinib (ARQ197) in children with relapsed or refractory solid tumors: a children’s oncology group study phase 1 and pilot consortium trial (ADVL1111). *Pediatr Blood Cancer.* (2017) 64:1–21. 10.1002/pbc.26565 28449393 PMC5657151

[184] Wagner AJ Goldberg JM Dubois SG Choy E Lee R Pappo A Tivantinib (ARQ 197), a selective inhibitor of MET, in patients with microphthalmia transcription factor-associated tumors: results of a multicenter phase 2 trial. *Cancer.* (2012) 118:5894–902. 10.1002/cncr.27582 22605650

[185] Yakes FM Chen J Tan J Yamaguchi K Shi Y Yu P Cabozantinib (XL184), a novel MET and VEGFR2 inhibitor, simultaneously suppresses metastasis, angiogenesis, and tumor growth. *Mol Cancer Ther.* (2011) 10:2298–308. 10.1158/1535-7163.MCT-11-0264 21926191

[186] Italiano A Mir O Mathoulin-Pelissier S Penel N Piperno-Neumann S Bompas E Cabozantinib in patients with advanced Ewing sarcoma or osteosarcoma (CABONE): a multicentre, single-arm, phase 2 trial. *Lancet Oncol.* (2020) 21:446–55. 10.1016/S1470-2045(19)30825-3 32078813 PMC8763616

[187] Chuk MK Widemann BC Minard CG Liu X Kim AR Bernhardt MB A phase 1 study of cabozantinib in children and adolescents with recurrent or refractory solid tumors, including CNS tumors: trial ADVL1211, a report from the children’s oncology group. *Pediatr Blood Cancer.* (2018) 65:1–7. 10.1002/pbc.27077 29693796 PMC6082380

[188] Akshintala S Widemann BC Barkauskas DA Hall D Reid JM Voss SD Phase 2 trial of cabozantinib in children and young adults with refractory sarcomas, Wilms tumor, and rare tumors: children’s oncology group study (ADVL1622). *J Clin Oncol.* (2021) 39:10010. 10.1200/JCO.2021.39.15_suppl.10010

[189] Perisa MP Storey M Streby KA Ranalli MA Skeens M Shah N. Cabozantinib for relapsed neuroblastoma: single institution case series. *Pediatr Blood Cancer.* (2020) 67:1–4. 10.1002/pbc.28317 32343886

[190] Mossé YP Lim MS Voss SD Wilner K Ruffner K Laliberte J Safety and activity of crizotinib for paediatric patients with refractory solid tumours or anaplastic large-cell lymphoma: a children’s oncology group phase 1 consortium study. *Lancet Oncol.* (2013) 14:472–80. 10.1016/S1470-2045(13)70095-0 23598171 PMC3730818

[191] Mossé YP Voss SD Lim MS Rolland D Minard CG Fox E Targeting ALK with crizotinib in pediatric anaplastic large cell lymphoma and inflammatory myofibroblastic tumor: a Children’s Oncology Group study. *J Clin Oncol.* (2017) 35:3215–21. 10.1200/JCO.2017.73.4830 28787259 PMC5617123

[192] Vassal G Faivre L Geoerger B Plantaz D Auvrignon A Coze C Crizotinib in children and adolescents with advanced ROS1, MET, or ALK-rearranged cancer: results of the AcSé phase II trial. *J Clin Oncol.* (2016) 34:11509. 10.1200/JCO.2016.34.15_suppl.11509

[193] Péron J Marreaud S Staelens D Raveloarivahy T Nzokirantevye A Flament J A multinational, multi-tumour basket study in very rare cancer types: the European organization for research and treatment of cancer phase II 90101 ‘CREATE’ trial. *Eur J Cancer.* (2019) 109:192–5. 10.1016/j.ejca.2018.12.013 30655100

[194] Schöffski P Wozniak A Leahy MG Aamdal S Rutkowski P Bauer S The tyrosine kinase inhibitor crizotinib does not have clinically meaningful activity in heavily pre-treated patients with advanced alveolar rhabdomyosarcoma with FOXO rearrangement: European organisation for research and treatment of cancer phase 2 trial. *Eur J Cancer.* (2018) 94:156–67. 10.1016/j.ejca.2018.02.011 29567632

[195] Broniscer A Jia S Mandrell B Hamideh D Huang J Onar-Thomas A Phase 1 trial, pharmacokinetics, and pharmacodynamics of dasatinib combined with crizotinib in children with recurrent or progressive high-grade and diffuse intrinsic pontine glioma. *Pediatr Blood Cancer.* (2018) 65:1–8. 10.1002/pbc.27035 29512900 PMC5980705

[196] Gibson EG Selvo NS Campagne O Gajjar A Stewart CF. Abstract 1357: population pharmacokinetic analysis of crizotinib in children with progressive/recurrent high-grade and diffuse intrinsic pontine gliomas. *Cancer Res* (2021) 81:1357L–1357. 10.1158/1538-7445.AM2021-1357 34586478 PMC8561710

[197] Balis FM Thompson PA Mosse YP Blaney SM Minard CG Weigel BJ First-dose and steady-state pharmacokinetics of orally administered crizotinib in children with solid tumors: a report on ADVL0912 from the children’s oncology group phase 1/Pilot consortium. *Cancer Chemother Pharmacol.* (2017) 79:181–7. 10.1007/s00280-016-3220-6 28032129 PMC5225209

[198] Greengard E Mosse YP Liu X Minard CG Reid JM Voss S Safety, tolerability and pharmacokinetics of crizotinib in combination with cytotoxic chemotherapy for pediatric patients with refractory solid tumors or anaplastic large cell lymphoma (ALCL): a children’s oncology group phase 1 consortium study (ADVL1212. *Cancer Chemother Pharmacol.* (2020) 86:829–40. 10.1007/s00280-020-04171-4 33095287 PMC7757912

[199] Gambacorti-Passerini C Orlov S Zhang L Braiteh F Huang H Esaki T Long-term effects of crizotinib in ALK-positive tumors (excluding NSCLC): a phase 1b open-label study. *Am J Hematol.* (2018) 93:607–14. 10.1002/ajh.25043 29352732 PMC5947833

[200] Kato S Jardim DL Johnson FM Subbiah V Piha-Paul S Tsimberidou AM Phase I study of the combination of crizotinib (as a MET inhibitor) and dasatinib (as a c-SRC inhibitor) in patients with advanced cancer. *Invest New Drugs.* (2018) 36:416–23. 10.1007/s10637-017-0513-5 29047029 PMC5908757

[201] Schöffski P Wozniak A Stacchiotti S Rutkowski P Blay JY Lindner LH Activity and safety of crizotinib in patients with advanced clear-cell sarcoma with MET alterations: European organization for research and treatment of cancer phase II trial 90101 “CREATE.”. *Ann Oncol.* (2017) 28:3000–8. 10.1093/annonc/mdx527 28950372 PMC5834120

[202] Schöffski P Wozniak A Kasper B Aamdal S Leahy MG Rutkowski P Activity and safety of crizotinib in patients with alveolar soft part sarcoma with rearrangement of TFE3: European organization for research and treatment of cancer (EORTC) phase II trial 90101 “CREATE.”. *Ann Oncol.* (2018) 29:758–65. 10.1093/annonc/mdx774 29216400

[203] Fisher MJ Shih CS Rhodes SD Armstrong AE Wolters PL Dombi E Cabozantinib for neurofibromatosis type 1–related plexiform neurofibromas: a phase 2 trial. *Nat Med.* (2021) 27:165–73. 33442015 10.1038/s41591-020-01193-6 PMC8275010

